# A critical role of platelet TGF-β release in podoplanin-mediated tumour invasion and metastasis

**DOI:** 10.1038/srep42186

**Published:** 2017-02-08

**Authors:** Ai Takemoto, Mina Okitaka, Satoshi Takagi, Miho Takami, Shigeo Sato, Makoto Nishio, Sakae Okumura, Naoya Fujita

**Affiliations:** 1 Division of Experimental Chemotherapy, Cancer Chemotherapy Center, Japanese Foundation for Cancer Research, Tokyo 135-8550, Japan; 2 Department of Computational Biology and Medical Sciences, Graduate School of Frontier Sciences, The University of Tokyo, Kashiwa, Chiba 277-8561, Japan; 3 Department of Thoracic Center, Cancer Institute Hospital, Japanese Foundation for Cancer Research, Tokyo 135-8550, Japan

## Abstract

The tumour microenvironment is critical for various characteristics of tumour malignancies. Platelets, as part of the tumour microenvironment, are associated with metastasis formation via increasing the rate of tumour embolus formation in microvasculature. However, the mechanisms underlying the ability of tumour cells to acquire invasiveness and extravasate into target organs at the site of embolization remain unclear. In this study, we reported that platelet aggregation-inducing factor podoplanin expressed on tumour cell surfaces were found to not only promote the formation of tumour-platelet aggregates via interaction with platelets, but also induced the epithelial-mesenchymal transition (EMT) of tumour cells by enhancing transforming growth factor-β (TGF-β) release from platelets. *In vitro* and *in vivo* analyses revealed that podoplanin-mediated EMT resulted in increased invasiveness and extravasation of tumour cells. Treatment of mice with a TGF-β-neutralizing antibody statistically suppressed podoplanin-mediated distant metastasis *in vivo*, suggesting that podoplanin promoted haematogenous metastasis in part by releasing TGF-β from platelets that was essential for EMT of tumour cells. Therefore, our findings suggested that blocking the TGF-β signalling pathway might be a promising strategy for suppressing podoplanin-mediated haematogenous metastasis *in vivo*.

The interaction between tumour cells and platelets was shown to play a role in malignant progression of tumours^1^,^2^,^3^. Tumour cell-platelet aggregates enhance the rate of tumour embolus formation in microvasculature by increasing the adhesiveness and bulkiness of the aggregates. Platelets also protect tumour cells from immunological assault and blood shear stress by coating their surface^4^,^5^,^6^. In experimental models, thrombocytopenia and antiplatelet agents were demonstrated to decrease the rate of lung metastasis in mice^7^,^8^. In clinical studies, long-term low-dose administration of the anti-platelet agent acetylsalicylic acid decreased the risk of distant metastasis^9^, further supporting the significant role of platelets in the development of haematogenous metastasis. Moreover, several factors secreted from the alpha-granules of activated platelets including transforming growth factor-β (TGF-β), vascular endothelial growth factor (VEGF) and platelet-derived growth factor (PDGF) enhance the motility of both tumour and vascular endothelial cells as well as the growth of tumour cells at secondary sites^10^,^11^,^12^. Thus, these factors released from platelets might contribute to tumour malignancy and are potential targets for the prevention of metastasis.

Several molecules, including glycoprotein Ib alpha (GPIbα)^13^, sialyl Lewis^x^/sialyl Lewis^a^ 14,15, Necl-5^16^, integrins^17^, thrombospondin-1^18^, high-mobility group box 1 (HMGB1)^19^ and podoplanin/Aggrus^20^, were found to potentially induce platelet aggregation and activation. Podoplanin is a type I transmembrane sialoglycoprotein that is frequently upregulated in a number of tumours including squamous cell carcinoma (SCC), mesothelioma, osteosarcoma, testicular germ cell tumour, glioblastoma and bladder tumour^21^,^22^,^23^,^24^,^25^,^26^. Podoplanin expression was found to correlate with the frequency of distant metastasis in bladder tumours and poor prognosis in brain tumours^25^,^26^. Additionally, increased podoplanin expression in cancer-associated fibroblasts (CAFs) and a positive correlation between CAF podoplanin expression and poor prognosis were demonstrated in lung adenocarcinoma^27^, invasive ductal carcinoma of breast and pancreas^28^,^29^ and melanoma^30^. Moreover, podoplanin expression was found in tumour-initiating cells, suggesting a pathological role for podoplanin in tumour progression^31^. Given that the forced expression of wild-type but not mutant podoplanin lacking platelet-aggregating ability led to the acquisition of metastatic ability of non-metastatic Chinese hamster ovary (CHO) cells and enhanced the rate of tumour arrest in the lung, the platelet aggregation-inducing activity of podoplanin was suggested to be directly linked to its ability to facilitate metastasis formation^32^. C-type lectin-like receptor 2 (CLEC-2), originally identified as a receptor for snake venom rhodocytin, was reported to be a counter-receptor for podoplanin^33^. Podoplanin binding to CLEC-2 induced platelet activation through Src family kinases Syk and phospholipase Cγ2 in platelets^34^. Furthermore, the phenotype of podoplanin knockout mice that included defective separation of lymphatic vessels from blood vessels during development was similar to that observed in mice deficient for CLEC-2^35^,^36^,^37^, confirming that CLEC-2 functions as a podoplanin receptor *in vivo*.

Although several groups reported the relationship between podoplanin expression and invasive phenotypes of tumor cells *in vitro*^38^,^39^, the exact molecular mechanisms underlying the acquisition of invasiveness and extravasation ability of tumour cells after podoplanin-mediated embolization in microvasculature remain unclear. Thus, we here investigated the role of podoplanin-mediated platelet aggregation in tumour invasiveness and extravasation.

## Results

### Podoplanin-mediated platelet aggregation promotes epithelial-mesenchymal transition in tumour cells

**Figure 1:**
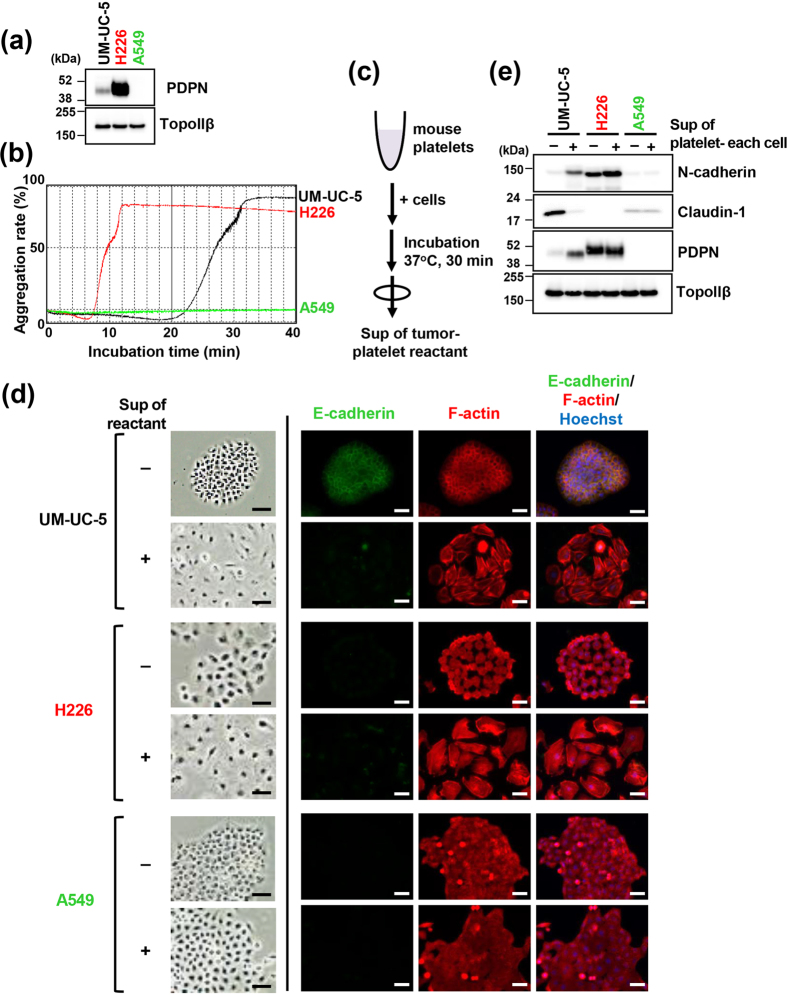
Podoplanin-mediated platelet aggregation promotes epithelial-mesenchymal transition in tumour cells. (**a**) Immunoblot analysis showing podoplanin expression in UM-UC-5 and H226 but not in A549 cells. TopoIIβ was used as a loading control. (**b**) UM-UC-5, H226 and A549 cells (5 × 10^4^ cells) were incubated with washed mouse platelets (5 × 10^7^ platelets/200 μl assay) suspended in Tyrode’s buffer containing 2% platelet-poor plasma and 250 μM CaCl_2_. Light transmittance of samples was measured to determine the aggregation rate using an aggregometer. (**c**) Schematic representation of collection of tumour-platelet reactant supernatants. (**d**,**e**) Morphological and physiological changes in cells after treatment with or without supernatants of tumor-platelet reactants for 48 h. (**d**) Images were captured using phase-contrast microscopy (left panels) or immunofluorescence microscopy (right panels) after staining for E-cadherin (green), F-actin (red; phalloidin) and nuclear DNA (blue; Hoechst 33342). Scale bars represent 50 μm. (**e**) Cellular lysates were immunoblotted with antibodies to N-cadherin, claudin-1, podoplanin (PDPN, clone D2–40) and TopoIIβ.

To elucidate the role of podoplanin-mediated tumour-induced platelet aggregation in tumour behaviour, we first investigated podoplanin-positive tumour cell lines for potential to induce platelet aggregation. Consistent with previous reports, podoplanin expression was detected in bladder SCC cell line UM-UC-5^26^ and malignant pleural mesothelioma cell line NCI-H226 (referred to as H226 from hereon)^21^, but not in lung adenocarcinoma cell line A549^40^ (Fig. 1a, Supplementary Fig. S1). In addition, podoplanin-positive UM-UC-5 and H226 cells but not A549 cells exhibited platelet aggregation activity (Fig. 1b). The dependency of this ability of UM-UC-5 cells on podoplanin was confirmed by inhibition of platelet aggregation by our established anti-podoplanin neutralizing monoclonal antibody (mAb) MS-1^40^ (Supplementary Fig. S2).

Platelets store several cytokines and growth factors in cytoplasmic granules which are released upon stimulation, leading to platelet activation. Thus, we next examined the effects of platelet-derived soluble factors released after podoplanin-induced platelet aggregation. To exclude the potential confounding effect of direct contact between tumour cells and platelets, we added supernatants of tumour cell-platelet reactants to naïve tumour cell cultures (Fig. 1c). We found that the morphology of UM-UC-5 and H226 cells, which appeared as clustered colonies in culture dishes, changed to a dispersed configuration after exposure to supernatants of tumour cell-platelet reactants (Fig. 1d). As such morphological change is one of the features of EMT, we next examined several EMT factors. Immunofluorescence staining revealed decreased E-cadherin expression and hyperplasia of actin stress fibers as observed by F-actin in UM-UC-5 cells (Fig. 1d). Moreover, immunoblotting showed increased expression of the mesenchymal marker N-cadherin and decreased expression of claudin-1, a critical component of tight junctions and an epithelial marker, in UM-UC-5 cells treated with supernatants of tumour cell-platelet reactants (Fig. 1e). We also found that podoplanin expression levels were also increased in these cells under the same conditions. Though H226 cells exhibited morphological changes similar to those observed in UM-UC-5 cells, such as the formation of actin stress fibres, following treatment with the supernatants of tumour cell-platelet reactants, EMT marker expression profile in H226 cells already indicated a mesenchymal-like status including low-level expression levels of E-cadherin and Claudin-1 and high-level expression level of N-cadherin. Therefore, changes observed in these EMT markers in H226 cells exposed to the supernatants of tumour cell-platelet reactants were subtler than those observed in UM-UC-5 cells. Conversely, A549 cells of epithelial origin did not exhibit morphological changes or EMT induction by treatment with supernatants of tumour cell-platelet reactants. These results indicated that released factors to the supernatant depending on platelet aggregation induce EMT on tumour cells.

### TGF-β release associated with podoplanin-induced platelet aggregation is critical for epithelial-mesenchymal transition

**Figure 2:**
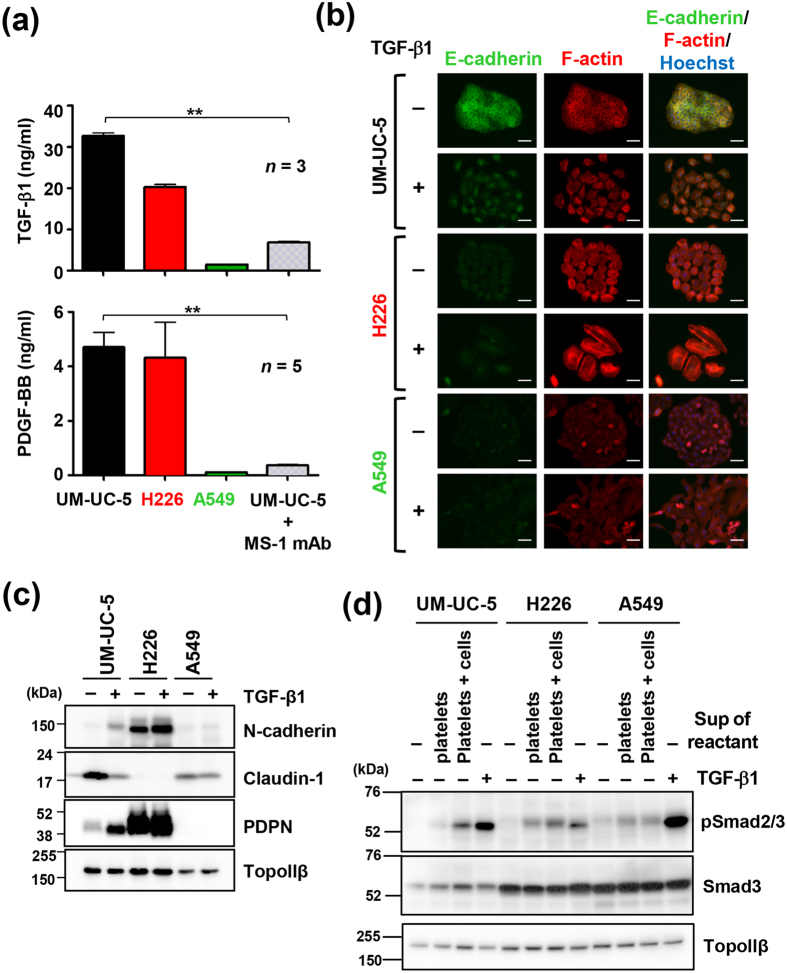
TGF-β released from platelets is critical for podoplanin-induced epithelial-mesenchymal transition. (**a**) Measurement of TGF-β1 or PDGF-BB concentrations in tumour-platelet reactants using Bio-Plex suspension array system. Error bars indicate standard deviation (SD). **P < 0.01 by Student’s *t* test. (**b**,**c**) Morphological and physiological changes in cells after treatment with or without 3 ng/ml recombinant TGF-β1 for 48 h. (**b**) Cells were stained for E-cadherin (green), F-actin (red; phalloidin) and nuclear DNA (blue; Hoechst 33342). Scale bars represent 50 μm. (**c**) Cell lysates were immunoblotted with antibodies to N-cadherin, claudin-1, podoplanin (PDPN, clone D2-40) and TopoIIβ. (**d**) Cells were either left untreated or treated with supernatants of platelets alone (platelets), supernatants of platelet–cell reactants (platelets + cells), or 3 ng/ml of recombinant TGF-β1 for 0.5 h. The cell lysates were immunoblotted with antibodies against phospho-Smad2/3 (pSmad2/3), Smad3, and TopoIIβ.

**Figure 3:**
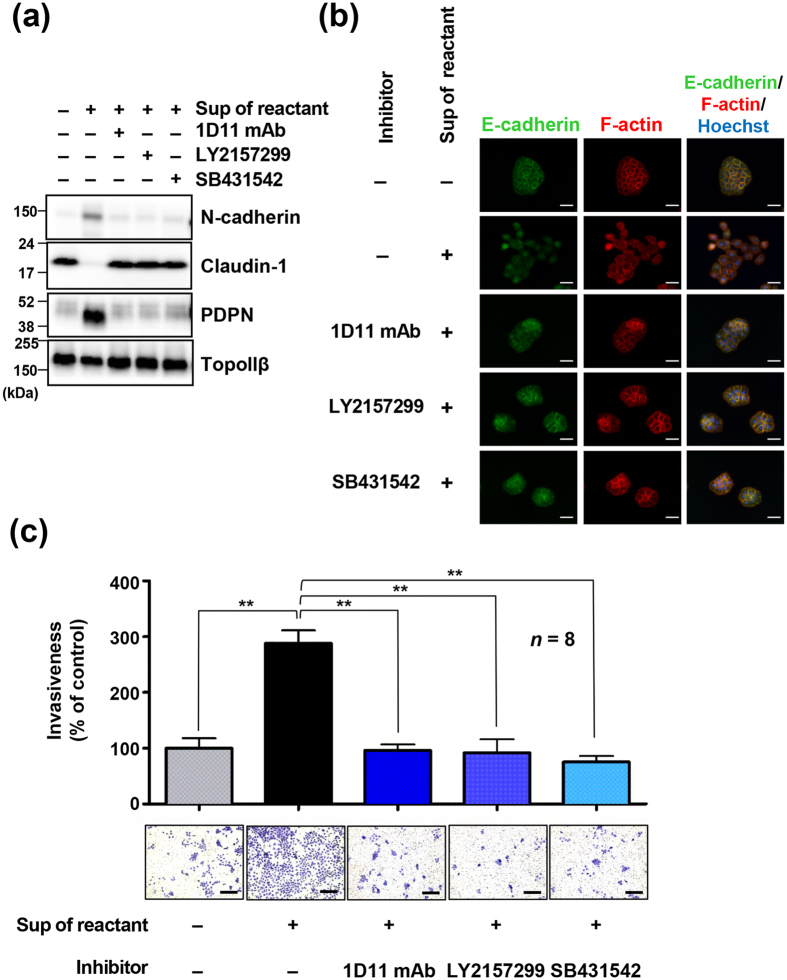
TGF-β/TGFβR signaling is involved in podoplanin-induced epithelial-mesenchymal transition in UM-UC-5 cells. (**a**–**c**) UM-UC-5 cells were treated with or without TGF-β1 neutralizing mAb (1D11 mAb) or TGFβR inhibitors (LY2157299 or SB431542) for 2 h, followed by incubation with supernatants of UM-UC-5-platelet reactants for 48 h. Morphological and physiological changes in treated cells were examined by immunoblotting (**a**), immunofluorescence staining (**b**) and invasion assay using a matrigel-coated transwell chambers (**c**). (**a**) Cell lysates were immunoblotted with antibodies to N-cadherin, claudin-1, podoplanin (PDPN, clone D2-40) and TopoIIβ. (**b**) Cells were stained for anti-E-cadherin (green), F-actin (red; phalloidin) and nuclear DNA (blue; Hoechst 33342). Scale bars represent 50 μm. (**c**) Cells were either left untreated or treated with supernatants of UM-UC-5-platelet reactants for 48 h. Next, 5 × 10^4^ UM-UC-5 cells were added to the upper chambers of matrigel-overlaid membranes. After incubation for an additional 48 h at 37 °C, cells migrating through the membranes were fixed and stained with crystal violet (lower panels; scale bars represent 200 μm). Optical density (OD) of crystal violet extracted from cells was measured at 540 nm and presented as a percentage of the OD values of control cells. All data are shown as means ± standard deviation (SD, n = 8). **P < 0.01 by the Mann-Whitney *U* test (upper panel).

Several platelet factors were shown to be released following activation^41^. Thus, we analyzed the levels of soluble factors in supernatants of tumour cell-platelet reactants using a Bio-Plex suspension array system for detection of multiple mouse cytokines. As seen in Fig. 2a and Supplementary Fig. S3, several soluble factors were at higher concentrations in the supernatants of UM-UC-5- and H226-platelet reactants than in the supernatants of A549-platelet reactants that were not aggregated. Importantly, the levels of these factors were reduced in the supernatants from anti-podoplanin mAb-treated UM-UC-5-platelet reactants in which platelet aggregation was suppressed (Fig. 2a; Supplementary Fig. S3). Specifically, the levels of TGF-β1 and PDGF-BB were much higher than other factors in the supernatants of UM-UC-5- and H226-platelet reactants (Fig. 2a; Supplementary Fig. S3). TGF-β is one of the most documented EMT inducers^42^. To examine the responsiveness of cells to TGF-β, cells were treated with purified active TGF-β1, which led to morphological changes including dispersion of cells and extensive actin stress fibers in UM-UC-5, H226 and A549 cells (Fig. 2b). Additionally, epithelial UM-UC-5 and A549 cells but not mesenchymal H226 cells exhibited decreased expression of E-cadherin and Claudin-1 and increased expression of N-cadherin, indicating the induction of EMT (Fig. 2b,c). A549 cells were responsive to TGF-β1; however, EMT was not induced by treatment with supernatants of A549-platelet reactants, implying that EMT induction was dependent on TGF-β released by aggregated platelets. Consistently, phosphorylation of Smad2/3, downstream of TGF-β signal activation, was not upregulated by treatment with supernatants of A549-platelet reactants compared to treatment with supernatants of only platelets, but it was upregulated by treatment with supernatants of UM-UC-5- or H226-platelet reactants, although Smad2/3 phosphorylation of all three cell lines was responsive to TGF-β1 treatment (Fig. 2d). We also used inhibitors of TGF-β signalling to determine the contribution of TGF-β1 to EMT of UM-UC-5 cells. As TGF-β signalling occurs through the formation of heterotetrameric receptor complexes of type I and type II TGF-β receptors (TGFβR1 and TGFβR2) by ligand binding, we treated UM-UC-5 cells with either a pan-TGF-β-neutralizing mAb (1D11) or TGF-β receptor inhibitors (LY2157299, targets both TGFβR1 and TGFβR2; SB431542, targets TGFβR1) and observed that morphological changes and EMT induced by supernatants of UM-UC-5 cell-platelet reactants were abolished (Fig. 3a,b). Conversely, addition of sunitinib inhibiting PDGF receptor did not have any inhibitory effects on morphological and EMT marker changes in UM-UC-5 cells (Supplementary Fig. S4).

EMT was shown to increase the invasiveness of tumor cells and was proposed to promote metastasis. Thus, we next assessed the effect of UM-UC-5-induced platelet aggregation on the invasion ability of UM-UC-5 cells and the contribution of TGF-β signal activation to invasiveness using matrigel-coated transwell chambers. Treatment with supernatants of UM-UC-5 cell-platelet reactants increased the invasiveness of UM-UC-5 cells, which was compromised by preincubation with the TGF-β mAb 1D11 or TGFβR inhibitors (Fig. 3c). These results indicated that TGF-β release on tumour cell-induced platelet aggregation and activation of the TGF-β signalling was critical for EMT and invasion of tumour cells.

### Podoplanin is essential for induction of TGF-β release into the supernatants of tumour cell-platelet reactants

**Figure 4:**
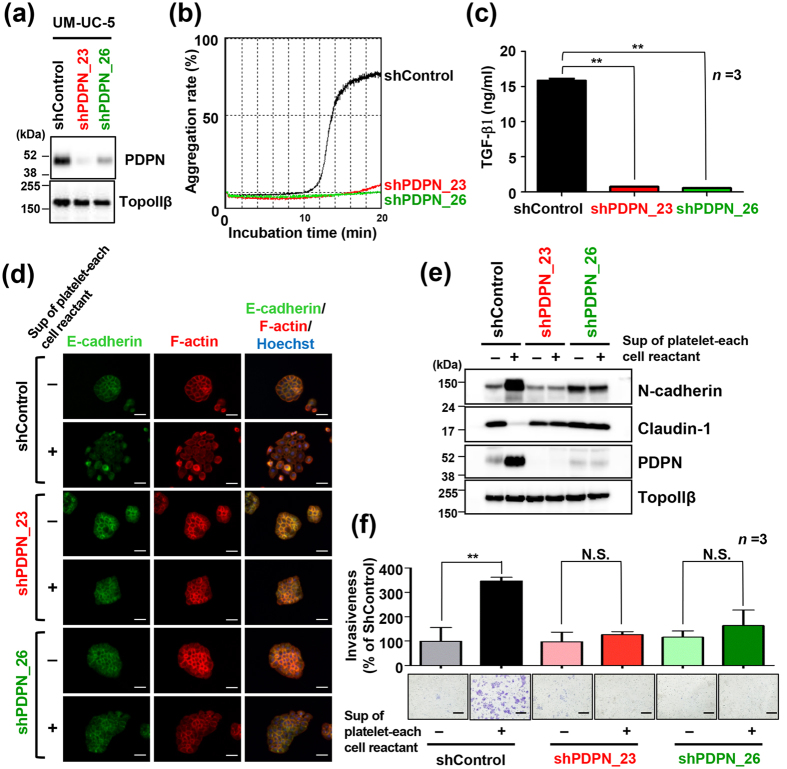
Podoplanin is necessary for TGF-β release from platelets and epithelial-mesenchymal transition. UM-UC-5 cells were infected with lentivirus containing shRNA targeting human podoplanin (shPDPN_23 and shPDPN_26) or control (shControl). Cells with stable knockdown of podoplanin were used in the experiments. (**a**) Immunoblot analysis of podoplanin expression in shPDPN_23, shPDPN_26 and shControl cells. TopoIIβ was used as a loading control. (**b**) ShPDPN_23, shPDPN_26 and shControl cells (5 × 10^4^ cells) were incubated with washed platelets (4 × 10^7^ platelets/200 μl) suspended in Tyrode’s buffer containing 2% platelet-poor plasma and 250 μM CaCl_2._ Light transmittance of samples was measured to determine the aggregation rate using an aggregometer. (**c**) TGF-β1 concentrations in tumour-platelet reactants were determined by enzyme-linked immunosorbent assay. All data are shown as means ± standard deviation (SD, n = 3). **P < 0.01 by the Mann-Whitney *U* test. (**d**,**e**) Morphological and physiological changes in shPDPN_23, shPDPN_26 and shControl cells after treatment with or without supernatants of tumour-platelet reactants for 48 h. (**d**) Cells were stained for E-cadherin (green), F-actin (red; phalloidin) and nuclear DNA (blue; Hoechst 33342). Scale bars represent 50 μm. (**e**) Cell lysates were immunoblotted with antibodies to N-cadherin, claudin-1, podoplanin (PDPN, clone D2-40) and TopoIIβ (**e**). (**f**) Cells were either left untreated or treated with supernatants of stable transfectant-platelet reactants for 48 h. Next, treated transfectants (5 × 10^4^/well) were added to the upper chambers of matrigel-overlaid membranes. After incubation for an additional 48 h at 37 °C, cells migrating through matrigel-overlaid membranes were fixed and stained with crystal violet (lower panels; scale bars represent 200 μm). Optical density (OD) of crystal violet extracted from cells was measured at 540 nm and presented as percentages of the OD values of supernatant-untreated shcontrol cells. All data are shown as means ± SD (n = 8). N.S., not significant. **P < 0.01 by the Mann-Whitney *U* test (upper panel).

To evaluate the significance of podoplanin in TGF-β release from tumour cell-platelet reactants, we established two UM-UC-5 cell lines in which podoplanin was knocked down, UM-UC-5/shPDPN_23 and UM-UC-5/shPDPN_26 (Fig. 4a). We confirmed that these cell lines showed attenuated platelet aggregation ability (Fig. 4b). Consistent with suppression of platelet aggregation induction by those cells, the levels of TGF-β1 in the supernatants of UM-UC-5/shPDPN_23- and UM-UC-5/shPDPN_26-platelet reactants were below the limit of detection by enzyme-linked immunosorbent assay (ELISA; Fig. 4c). Furthermore, addition of supernatants of the podoplanin-knocked down cell-platelet reactants failed to induce morphological changes, EMT (Fig. 4d,e) or invasiveness of each cells (Fig. 4f), even if those cells were responsive to TGF-β1 (Supplementary Fig. S5a) and rescued by TGF-β1-supplemented supernatants (Supplementary Fig. S5b). In a mouse metastasis model, haematogenous metastasis to the lung was suppressed by podoplanin knockdown in UM-UC-5 cells that were inoculated to the mice (Supplementary Fig. S6). These results indicated that podoplanin was essential for TGF-β release from platelets and subsequent EMT, invasion and eventual metastasis.

### TGF-β enhances the rate of tumour extravasation *in vivo*

**Figure 5:**
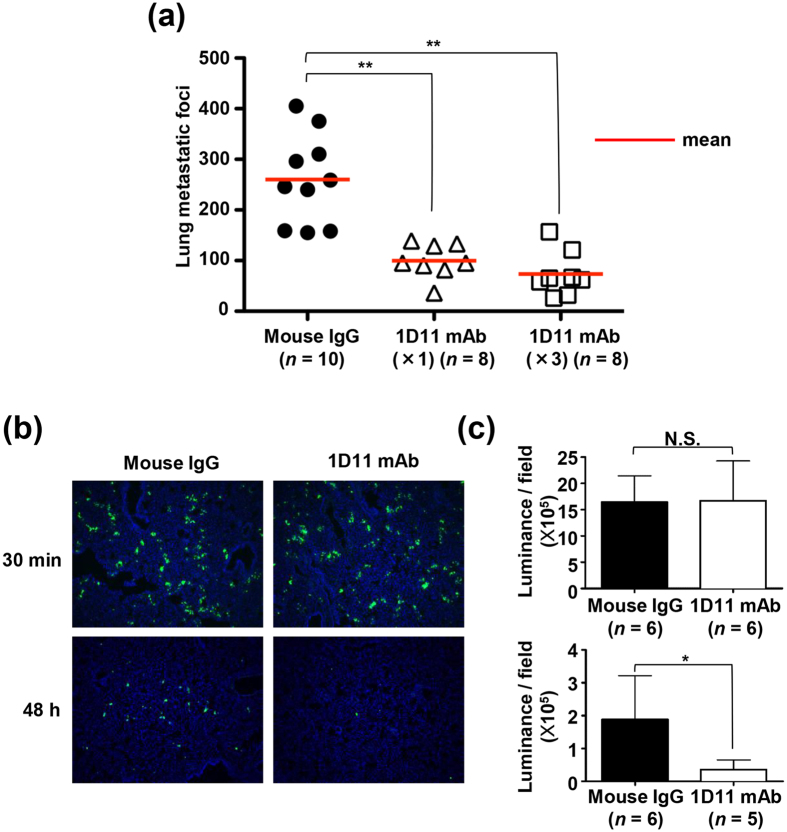
Neutralization of TGF-β attenuates tumour extravasation and pulmonary metastasis. (**a**) Mouse control IgG (Mouse IgG) or TGFβ neutralizing mAb (clone 1D11) (100 μg/mouse) was administrated by the intravenous (i.v.) route to 5-week-old male CB17/Icr-Prkdc^scid^/CrlCrlj mice 1 h before i.v. inoculation of UM-UC-5 cell suspensions (1.0 × 10^6^/mouse). In some experiments, 1D11 mAb was administrated 2 and 4 days after tumor cell inoculation (1D11 mAb (x3)). Mice were euthanized 30 days after cell inoculation and metastatic foci on the lung surface were counted. Bars represent mean values. **P < 0.01 by the Mann-Whitney *U* test. (**b**) Mouse control IgG (Mouse IgG) or TGFβ neutralizing mAb (clone 1D11) (100 μg/mouse) was administrated to 5-week-old male CB17/Icr-Prkdc^scid^/CrlCrlj mice 1 h before tumour inoculation. Calcein-AM-labeled UM-UC-5 cell suspensions were then inoculated (1.0 × 10^6^/mouse) into mice. After 30 min or 48 h after i.v. tumour inoculation, mice were euthanized and frozen lung sections were fixed and stained by Hoechst 33342. Representative merged images of calcein-AM-labeled UM-UC-5 cells (green) stained for nuclear DNA (blue) are shown. (**c**) The fluorescence intensity of calcein-AM was measured using BioRevo BZ-9000. N.S., not significant. *P < 0.05 by the Mann-Whitney *U* test.

To examine the contribution of TGF-β release during podoplanin-mediated platelet aggregation on metastasis efficacy, we first analysed the effect of 1D11 mAb on haematogenous metastasis formation by UM-UC-5 cells. In our mouse model, metastatic foci on the lung surfaces that were established by intravenous (i.v.) inoculation of UM-UC-5 cells via the tail vein were decreased by a single dose of 1D11 mAb 1 hr prior to the inoculation (Fig. 5a). However, we observed that two additional doses of 1D11 mAb at 2 and 4 days after i.v. UM-UC-5 inoculation did not lead to a further decrease in metastatic foci formation. This result suggested that TGF-β contributed to an early step in metastasis formation following the entry of tumour cells into circulation. To gain a more detailed insight into the role of TGF-β during metastasis, we analysed the cells trapped in lung tissue for the number of surviving tumour cells soon after the i.v. inoculation of UM-UC-5 cells in an *in vivo* model of metastasis. Briefly, control IgG or 1D11 mAb was administrated by i.v. injection via the tail vein of mice, followed by i.v. inoculation of calcein AM-labelled UM-UC-5 cells after 1 h. Mice were euthanized at 30 min or 48 h after inoculation of cells, and the lungs were excised. Sectioned lung specimens were fixed and the number of UM-UC-5 cells were determined using calcein AM labelling. As presented in Fig. 5b and c, the number of UM-UC-5 cells trapped in lungs at 30 min was similar between control IgG- and 1D11-treated mice (*P* = 0.631 by Mann-Whitney *U* test). In contrast, administration of 1D11 mAb significantly reduced the number of UM-UC-5 cells in lungs at 48 h after cell inoculation (*P* = 0.0173 by Mann-Whitney *U* test) (Fig. 5b,c), suggesting that TGF-β contributed to the immune evasion of tumour cells potentially via enhanced extravasation following EMT induction. However, there is also a possibility that TGF-β affects the pathways related to cell survival, apoptosis, etc., after tumour embolization. To confirm that mechanism by which platelets promoted metastasis through TGF-β release and EMT induction in podoplanin-positive epithelial tumours extended to other tumour, we analysed patient-derived lung SCC cell lines expressing podoplanin (Supplementary Fig. S7a). SCC-015 cells, a cell line established at our laboratory that can haematogenously induce metastatic foci in lung tissue, induced podoplanin-dependent platelet aggregation and led to TGF-β1 release (Supplementary Fig. S7b,c). Moreover, Smad3 phosphorylation and morphological changes such as EMT were observed in SCC-015 cells treated with supernatants of SCC-015-platelet aggregates as well as those treated with TGF-β1 (Supplementary Fig. S7d–f; Fig. S8). Furthermore, the observed EMT-like morphology was suppressed by pretreatment with 1D11 mAb or LY2157299 (Supplementary Fig. S7d). In contrast, other established podoplanin-positive SCC cell lines that did not metastasize did not undergo EMT following TGF-β1 treatment (Supplementary Fig. S8).

## Discussion

Numerous growth factors and cytokines stored in platelets are released during platelet activation not only by physiological agonists such as thrombin but also by tumour cells. These platelet factors have a physiological role in hemostasis and vessel stability, whereas in the presence of a tumour, they contribute to its growth, survival, invasion, and angiogenesis^1^; however, the exact effect of platelets on tumour cells is unclear. In this study, we found that podoplanin-positive tumour cells induced platelet aggregation and that growth factors and cytokines were released during platelet aggregation through the podoplanin–platelet interaction. As previously reported, this podoplanin-mediated pathway is different from the physiological situation in platelet activation^33^, although it also results in the release of platelet factors (Fig. 2a; Supplementary Fig. S3).

We fo6und that TGF-β knockdown in UM-UC-5 cells did not affect the level of TGF-β released on platelet aggregation and that platelets seemed to contain much more TGF-β than UM-UC-5 cells when they were compared at the ratio used in the platelet aggregation assay. These results suggested that TGF-β released on platelet aggregation was mainly derived from platelets and not from UM-UC-5 cells (Supplementary Fig. S9).

In this study, we demonstrated that TGF-β played a crucial role in the induction of EMT in tumour cells. Labelle *et al*. found that factors released from activated platelets by the physiological coagulation factor thrombin were not sufficient for EMT induction in breast carcinoma and that direct contact with platelets through the NF-κB pathway activation was also required^10^. As we did not observe NF-κB pathway activation resulted in MCP-1/CCL2 release in UM-UC-5 cells in direct contact with platelets (Supplementary Fig. S10), platelet-mediated effects such as EMT were likely cell type-specific. A previous study showed that factors released by platelets and subsequent phenotypes were stimulus-dependent^43^. Tumour-induced platelet activation might be distinct from physiological platelet activation in TGF-β signalling of tumour cells. In addition, podoplanin is rarely expressed in breast cancer; thus, platelet activation by podoplanin-expressing tumours might be qualitatively different from that induced by podoplanin-negative tumours.

Platelets contain 40–100 times more TGF-β than other non-neoplastic cells^44^; however, TGF-β released by platelets is in an almost inactive (latent) form in complex with latency-associated peptide^45^. In fact, we found that the ratio of active TGF-β to total TGF-β (~30 ng/ml, Fig. 2a) released by UM-UC-5-platelet reactants was approximately 1% (estimated as ~0.3 ng/ml) by a bioassay using a mink lung epithelial cell line, Mv1Lu (Supplementary Fig. S11). In addition, EMT induction in UM-UC-5 cells required a higher level of active TGF-β (~0.3 ng/ml) than that contained in supernatant diluted 4–5 times in co-cultures (estimated active TGF-β1: less than 0.1 ng/ml) (Supplementary Fig. S12). Therefore, there could be a mechanism for TGF-β activation. Activation of TGF-β is suggested to be mediated by intermediate molecules including integrins, matrix metalloproteinases (MMPs), plasmin and reactive oxygen species^46^. Thus, proteases released from platelets or adhesion to tumour cell membrane factors might be involved in TGF-β activation in the co-culture system used in the present study.

In the present study, we clearly demonstrated that podoplanin-positive tumour cells acquired high invasive ability through EMT induction following TGF-β release from activated platelets. Martín-Villar *et al*. previously showed that podoplanin induced EMT by activating RhoA via direct interaction between the cytoplasmic region of podoplanin and ERM family proteins, i.e. ezrin, radixin and moesin^38^. Conversely, Wicki *et al*. reported that podoplanin overexpression promoted invasion ability of MCF-7 breast cancer cells and enhanced tumour formation and metastatic ability without inducing EMT^39^. In the present study, neither expression levels of EMT marker proteins (Fig. 4d,e) nor invasion ability (Fig. 4f) of UM-UC-5 cells were altered by podoplanin knockdown. Expression of the three ERM proteins are tissue-specific. For example, ezrin is found primarily in epithelial cells, whereas endothelial cells express moesin^47^. The difference in expression patterns of ERM proteins might affect EMT and podoplanin-dependent invasion. Increased podoplanin expression following treatment with TGF-β or supernatants of tumour-platelet reactants might lead to further acceleration of tumour malignancy through enhanced tumour-platelet interaction. Depending on the cell type, podoplanin expression affects cellular EMT status and invasion activity. In such cells, TGF-β induces EMT/invasion possibly through an increase in podoplanin expression^48^.

Peripheral blood TGF-β levels were reported to be higher in patients with lung, gastric, oesophagus, colon and brain tumours than in healthy volunteers^49^. Although upregulation of TGF-β in peripheral blood were suggested to be mediated by either tumour or tumour stromal cells, our findings indicated that tumour-platelet interaction as a new potential source for the increased levels of these factors in blood. In fact, TGF-β levels in blood tended to increase 1 h after i.v. inoculation of UM-UC-5 cells in mice (Supplementary Fig. S13). As shown by several previous studies, TGF-β act as enhancers of cancer metastasis^50^ and prevention of platelet aggregation could be critical for suppression of the release of these factors into the tumour microenvironment and blood.

EMT is widely recognized for its significant role in infiltration of tumour cells into neighbouring tissues, intravasation and extravasation. Moreover, Yu *et al*. demonstrated the presence of platelet-mesenchymal circulating tumour cell clusters in breast cancer patients and suggested that mesenchymal transformation of epithelial cells was mediated by TGF-β released from platelets^51^. In the current study, we showed that administration of an anti- TGF-β 1D11 mAb decreased the number of both lung-trapped UM-UC-5 cells and lung metastatic foci in an experimental metastasis model (Fig. 5). These inhibitory effects of 1D11 mAb were likely mediated by the inhibition of EMT of UM-UC-5 cells in peripheral blood and their subsequent extravasation. As TGF-β was also shown to suppress the antitumor function of natural killer cells by downregulating NKG2D immunoreceptor^4^, administration of 1D11 mAb might restore antitumor immunity mounted by the host. In addition, podoplanin knockdown resulted in suppression of pulmonary metastasis of UM-UC-5 cells, implicating both TGF-β and podoplanin as potential therapeutic targets for development of antimetastatic therapeutics.

As shown in Fig. 5, administration of 1D11 mAb 1 h prior to tumour cell inoculation significantly reduced the number of lung metastatic foci. In this short-term metastasis model (Fig. 5b,c), 1D11 mAb injection reduced the number of surviving tumour cells 48 h but not 30 min after their i.v. inoculation. This finding suggested that TGF-β might play a role in the extravasation step. In contrast, we observed that the administration of podoplanin-neutralizing antibody MS-1 reduced the number of lung-trapped tumour cells after only 30 min, suggesting that podoplanin promoted the trapping of tumour cells in lung^26^. In summary, our findings suggest that podoplanin-dependent platelet aggregation promotes tumour embolization and extravasation via EMT induction by TGF-β release from platelets.

## Methods

### Cell culture and reagents

UM-UC-5 human bladder SCC cell line was purchased from the Health Protection Agency (Salisbury, UK) and cultured in minimum essential medium (MEM, Wako, Osaka, Japan) containing 1× nonessential amino acids (NEAA, Wako, Osaka, Japan) and 10% fetal bovine serum (FBS). NCI-H226 human malignant pleural mesothelioma cell line was purchased from the American Type Culture Collection (ATCC) and cultured in RPMI 1640 medium (Wako) containing 10% FBS. A549 human lung adenocarcinoma cell line purchased from ATCC was cultured in Dulbecco’s modified Eagle’s medium (DMEM, Wako) containing 10% FBS. 293FT was purchased from Thermo Fisher Scientific (Waltham, MA, USA) and cultures in DMEM high glucose medium (Wako) containing NEAA and 10% FBS. The UM-UC-5 cell lines that stably expressed shRNAs targeting human podoplanin (UM-UC-5/shPDPN_23 and UM-UC-5/shPDPN_26) or control sequence (UM-UC-5/shControl) were established by lentiviral infection and cultured in MEM containing 10% FBS and 2 μg/ml puromycin (Life Technologies, Carlsbad, CA, USA). 1D11.16.8 hybridoma cells producing TGF-β-neutralizing mAb (1D11) were purchased from the ATCC and cultured in DMEM containing 0.1 mM NEAA and 10% FBS. TGFβR inhibitors LY2157299 and SB431542 were purchased from Shanghai Biochemical (Shanghai, China).

### Animals

Female Jcl:ICR mice were purchased from Clea Japan (Tokyo, Japan). Female Crl:CD1 (ICR) mice and male CB17/Icr-*Prkdc*^*scid*^/CrlCrlj mice were purchased from Charles River Laboratories Japan (Kanagawa, Japan). All animal procedures were performed using protocols approved by the Japanese Foundation for Cancer Research Animal Care and Use Committee in accordance with the relevant guidelines and regulations.

### Immunoblotting

Cells were lysed in lysis buffer (0.1 M Tris-HCl, pH 7.5, 1% sodium dodecyl sulfate [SDS], 10% glycerol) and boiled at 100 °C for 5 min and supernatants were collected after centrifugation at 20,000 *g* for 5 min. Protein concentrations were determined with BCA protein assay (Thermo Fisher Scientific) and 30 μg total protein were loaded on SDS-polyacrylamide gels (5–20% gradient) for electrophoresis separation. Proteins were then transferred to Immobilon-P polyvinylidene fluoride (PVDF) membranes (Merck Millipore, Darmstadst, Germany) and immunoblotted with antibodies against human podoplanin (D2–40; AbD Serotec, Kidlington, UK or Dako, Glostrup, Denmark), N-cadherin (Cell Signaling Technology, Danvers, MA, USA), Claudin-1 (Cell Signaling Technology), pSmad3 (Cell Signaling Technology), pSmad2/3 (Cell Signaling Technology), Smad3 (Cell Signaling Technology), TopoIIβ (clone 40; BD Transduction Laboratories, Washington, DC, USA) and β-actin (clone AC-15; Santa Cruz, Dallas, TX, USA). ECL Prime Western Blotting Detection Reagent from GE Healthcare (Chanford, UK) and LAS-3000 mini (Fujifilm, Tokyo, Japan) or Amersham Imager 600 (GE Healthcare) were used for detection of signals.

### Immunofluorescence staining

Cells plated onto coverslips were cultured for the indicated time periods, fixed with 4% paraformaldehyde in phosphate-buffered saline (PBS), pH 7.4 for 15 min and permeabilized with 0.1% TritonX-100 in PBS for 5 min. Anti-E-cadherin (clone: HECD-1, Takara Bio, Shiga, Japan) and rhodamine- or Texas red-conjugated phalloidin (Life Technologies) were diluted in PBS containing 2% BSA as primary antibodies and cells were incubated for 90 min. Alexa Fluor 488-conjugated anti-mouse IgG (Life Technologies) was used as the secondary antibody and nuclei were stained with 1 μg/ml Hoechst 33342 (Life Technologies) for 5 min. Images of cells were captured by BioRevo BZ-9000 (KEYENCE, Osaka, Japan).

### Platelet aggregation assay

Murine whole blood was drawn by cardiac puncture from Jcl:ICR or Crl:CD1 (ICR) mice terminally anesthetized with chloroform or sevoflurane into syringe containing sodium citrate at a final concentration of 0.38% or heparin at a final concentration of 10 units/ml (Mochida Pharmaceutical, Tokyo, Japan). Platelet-rich plasma (PRP) was collected from supernatants of murine whole blood by centrifugation at 110 *g* for 8 min. Washed platelets were prepared from PRP pellets by centrifugation at 500 *g* for 10 min, followed by a final wash with modified Tyrode’s solution (137 mM NaCl, 11.9 mM NaHCO_3_, 0.4 mM Na_2_HPO_4_, 2.7 mM KCl, 1.1 mM MgCl_2_, 5.6 mM glucose)^52^. Washed platelets were resuspended in modified Tyrode’s solution containing 2% platelet-poor plasma (PPP) at a concentration of 2–3 × 10^8^ platelets/ml. Before experiments, 250 μM CaCl_2_ was added to platelet suspensions. Platelet aggregation assay using a platelet aggregometer (MCM Hema Tracer 313 M; SSR Engineering, Kanagawa, Japan) was performed as previously described^12^. Briefly, 5 × 10^4^ tumour cells in 10 μl PBS were added to the platelet suspension as platelet aggregation inducers. In some experiments, UM-UC-5 or SCC-015 cells were incubated with 1–100 μg/ml anti-podoplanin mAb (MS-1 or PG4D2) or control mouse IgG2a (Sigma Aldrich) for 30–45 min on ice prior to the platelet aggregation assay.

### Preparation of platelet aggregation reactant supernatants

Platelets and cells were prepared as described for the platelet aggregation assay. After incubation of platelets with cells for 20–30 min at 37 °C, the reactants were centrifuged at 20,000 *g* for 5 min. Supernatants were then collected for cell treatments, Bio-Plex assay, or ELISA.

### Bio-Plex assay

Bio-Plex Pro^TM^ Mouse Cytokine GII 9-Plex and Bio-Plex Pro^TM^ TGF-β 3-Plex assays were purchased from Bio-Rad (Hercules, CA, USA). Supernatants of tumour cell-platelet reactants were analysed according to the manufacturer’s protocol. Signal detection by measurement of fluorescence intensity in each well and data analysis were performed using the Bio-Plex 200 System and Bio-Plex Manager 5.0 (Bio-Rad).

### Enzyme-linked immunosorbent assay

Quantikine Mouse/Rat/Porcine/Canine TGF-β1 immunoassays were purchased from R&D Systems (Minneapolis, MN, USA). Growth factors in supernatants of tumour cell-platelet reactants were measured according to the manufacturer’s protocol.

### Invasion assay

BD BioCoat^TM^ matrigel invasion chambers (pore size, 8.0 μm; BD Biosciences) or chemotaxicell membranes (pore size, 8.0 μm; Kurabo, Osaka, Japan) coated with BD matrigel basement membrane matrix (BD Biosciences) were used for the invasion assay. UM-UC-5 cells seeded on culture dishes were incubated with or without supernatants of tumour-platelet reactants or TGF-β1 (3 ng/ml) for 48 h. Cells were then harvested and counted to adjust cell numbers. Next, 5 × 10^4^ cells in 0.5 ml serum-free culture medium were added to the upper chambers, whereas 0.75 ml culture medium containing 10% FBS was added to the lower wells. After incubation for 48 h at 37 °C, cells that transferred to the lower chambers through the transmembrane were fixed with 4% paraformaldehyde and stained with 0.1% crystal violet. Cells remaining on the upper surface of the membranes were removed by wiping with cotton swabs. Dye extraction was achieved by adding 0.4 ml of 1 mM HCl in 30% ethanol into the wells and soaking transwell chambers. The absorbance was measured at 540 nm.

### ShRNA knockdown of podoplanin

Lentiviral expression vectors encoding shRNAs targeting human podoplanin (TRCN0000061923: shPDPN_23 and TRCN0000061926: shPDPN_26) and control vector (SHC001: shControl) were purchased from Sigma-Aldrich. Lentiviral vectors were cotransfected with the ViraPower^®^ packaging mix (Life Technologies) into 293FT cells using Lipofectamine 2000 (Life Technologies), followed by incubation for 48 h. Collected culture media containing lentiviral particles were added to the medium of UM-UC-5 cells, which were then maintained with 2 μg/ml puromycin to select for transduced cells.

### Experimental pulmonary metastasis

A total of 1.0 × 10^6^ UM-UC-5 cells/mouse were suspended in Hanks’ balanced salt solution (HBSS, Invitrogen) and injected intravenously into the lateral tail vein of 5-week-old male CB17/Icr-*Prkdc*^*scid*^/CrlCrlj mice. Mouse control IgG or anti-TGF-β mAb (1D11) (100 μg/mouse) was intravenously administrated 1 h before cell inoculation. Additionally, in some experiments, 1D11 mAb was administrated two more times after tumour cell inoculation for a total of three times. After approximately 30 days, mice were euthanized and resected lungs were stained with saturated picric acid solution. Metastatic foci on lung surfaces were then counted.

### Lung trap assay

UM-UC-5 cells stripped by trypsin treatment were incubated with 2 μM calcein-AM (Nacalai Tesque, Kyoto, Japan) for 30 min at 37 °C. After washing with PBS, cells were suspended in HBSS and intravenously injected (1.0 × 10^6^ cells/mouse) into the lateral tail vein of 5-week-old male CB17/Icr-*Prkdc*^*scid*^/CrlCrlj mice. Mouse control IgG or anti-TGF-β (1D11) mAb (100 μg/mouse) was intravenously administrated into the lateral tail vein of mice 1 h before the inoculation of cell suspension. After 30 min or 48 h after cell inoculation, mice were euthanized and resected lungs were frozen in Tissue-Tek OCT embedding compound (Sakura Finetek, Tokyo, Japan). Frozen lung specimens that were 10 μm thick were fixed with 4% paraformaldehyde and counterstained with Hoechst 33342. Fluorescence intensity of calcein-AM-labeled micrometastatic foci in each image was quantified using a BioRevo BZ-9000.

### Statistical analysis

The Mann-Whitney *U* test or Student’s *t* test was performed to determine the statistical significance of results. All statistical tests were two-sided. Significant *P* values were shown as *P < 0.05 and **P < 0.01.

### Establishment of patient-derived lung squamous cell carcinoma cell lines

Clinical lung squamous cell carcinoma specimens were obtained from patients after obtaining informed consent for biological analyses in accordance with the protocols approved by the institutional review board (IRB) of Japanese Foundation for Cancer Research (JFCR). Established patient-derived lung squamous cell carcinoma line SCC-015, SCC-037, and SCC-058 was cultured in StemPro^®^ hESC SFM (Thermo Fisher Scientific) supplemented with 10 μM Rho-associated kinase inhibitor Y27632 (AdooQ BioScience, Irvine, CA, USA) and 1x antibiotic-antimycotic mixed solution (Nacalai Tesque) on a collagen-coated dishes (IWAKI, Tokyo, Japan), and used for each *in vitro* analyses. All experiments were performed in accordance with relevant guidelines and regulations and following the protocols approved by the IRB of JFCR.

## Additional Information

**How to cite this article**: Takemoto, A. *et al*. A critical role of platelet TGF-β release in podoplanin-mediated tumour invasion and metastasis. *Sci. Rep.* **7**, 42186; doi: 10.1038/srep42186 (2017).

**Publisher's note:** Springer Nature remains neutral with regard to jurisdictional claims in published maps and institutional affiliations.

## Supplementary Material

Supplementary Figures
