# Supplementary material for: A critical role of platelet TGF-β release in podoplanin-mediated tumour invasion and metastasis
Source: Sci Rep. 2017 Feb 8;7:42186. doi: 10.1038/srep42186 (PMC5297242; doi:10.1038/srep42186)
Supplement: Supplementary Figures [file srep42186-s1.pdf]

A critical role of platelet TGF-β release in podoplanin-mediated tumour invasion and metastasis

Ai Takemoto[1], Mina Okitaka[1,2], Satoshi Takagi[1], Miho Takami[1], Shigeo Sato[1], Makoto Nishio[3], Sakae Okumura[3], and Naoya Fujita[1,2,*]

[1]Division of Experimental Chemotherapy, Cancer Chemotherapy Center, Japanese Foundation for Cancer Research, Tokyo 135-8550, Japan
[2]Department of Computational Biology and Medical Sciences, Graduate School of Frontier Sciences, The University of Tokyo, Kashiwa, Chiba 277-8561, Japan
[3]Department of Thoracic Center, Cancer Institute Hospital, Japanese Foundation for Cancer Research, Tokyo 135-8550, Japan

***Corresponding Author:** Naoya Fujita, Ph.D., Cancer Chemotherapy Center, Japanese Foundation for Cancer Research, Tokyo 135-8550, Japan.
Phone: +81-3-3570-0468; FAX: +81-3-3570-0484; E-mail: naoya.fujita@jfcr.or.jp

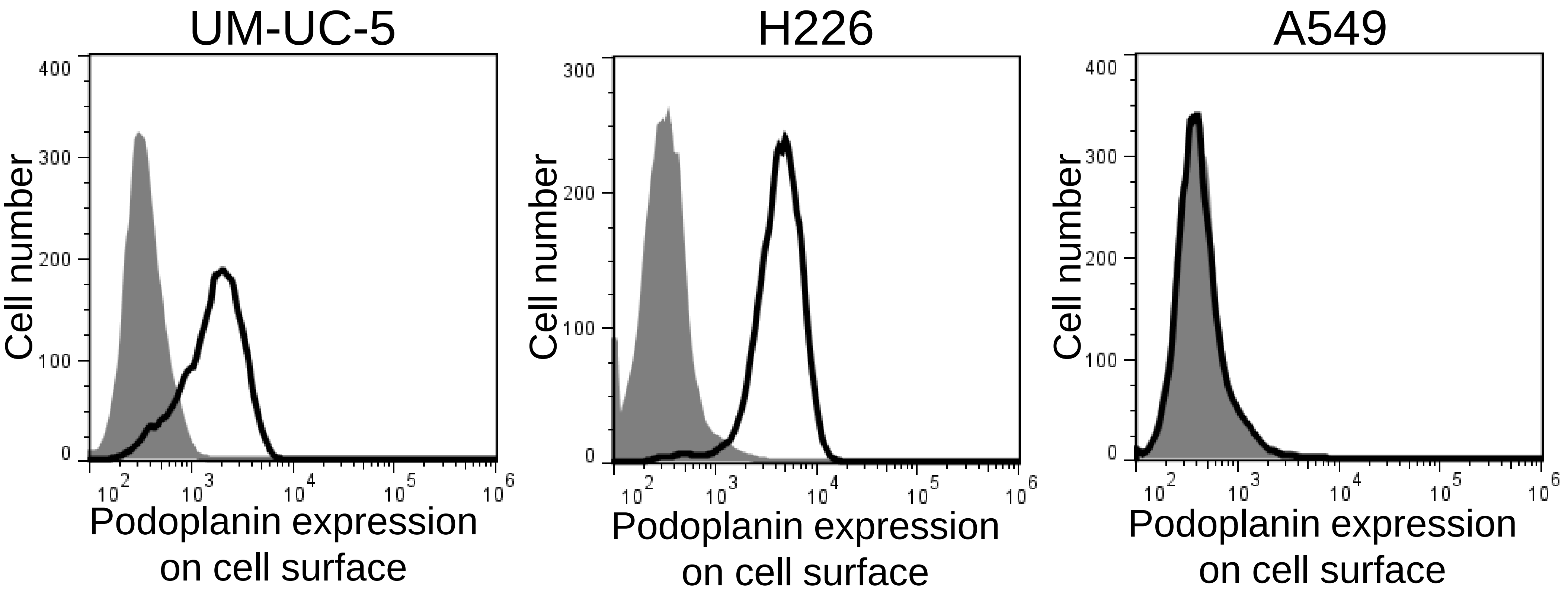

Figure S1. Podoplanin expression on cell surfaces of UM-UC-5, H226, and A549. UM-UC-5, H226, and A549 cells were subjected to flow cytometric analysis to detect podoplanin expression on the cell surface. Open areas indicate the cells detected by anti-podoplanin mAb (D2-40) as a primary detection antibody and secondary anti-mouse IgG conjugated Alexa 488. Filled areas are background detected only by secondary anti-mouse IgG conjugated Alexa 488 without D2-40. Podoplanin expression on UM-UC-5 and H226 cell surfaces was confirmed.

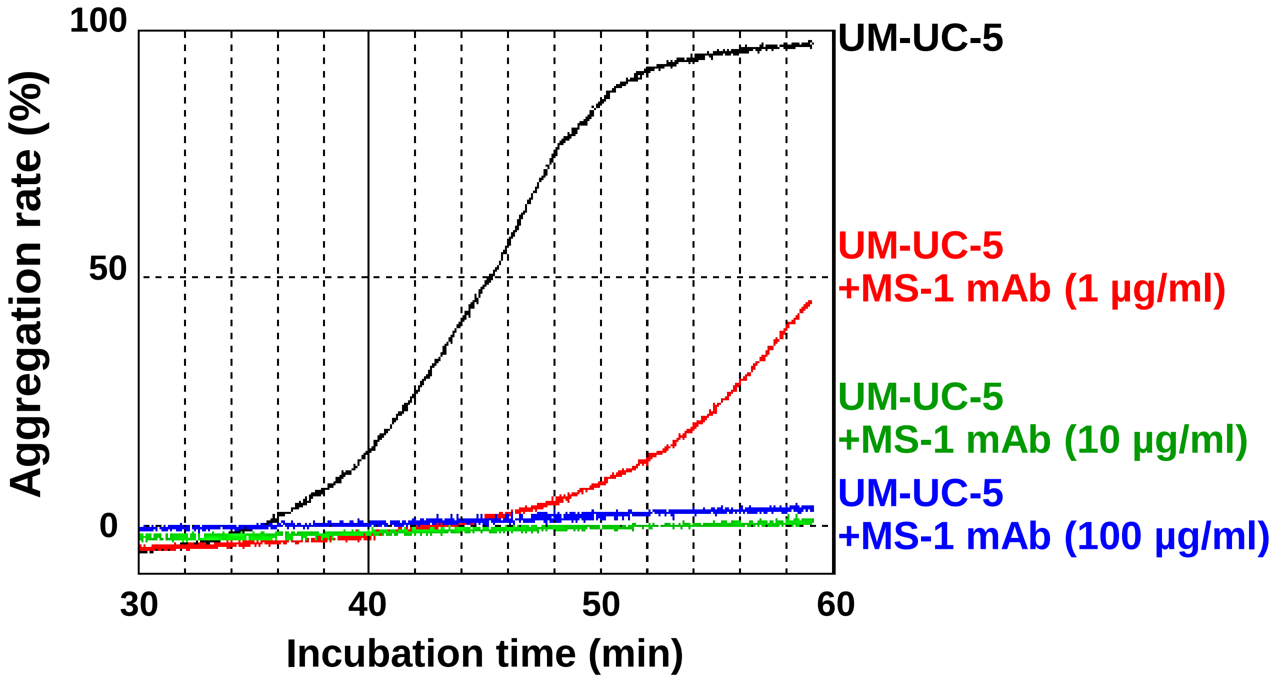

Figure S2. Podoplanin is involved in UM-UC-5-induced platelet aggregation. UM-UC-5 cells (5×10^4 cells/assay) were incubated with anti-podoplanin neutralizing MS-1 mAb (1, 10 or 100 µg/ml) on ice for 30 min, followed by incubation with washed platelets prepared from mouse whole blood (4×10^7 platelets/200 µl assay) in Tyrode's buffer containing 2% platelet-poor plasma and 250 µM $CaCl_2$. Light transmittance of samples was measured to determine aggregation rate using an aggregometer. Aggregation rate (%) compared to control buffer is shown.

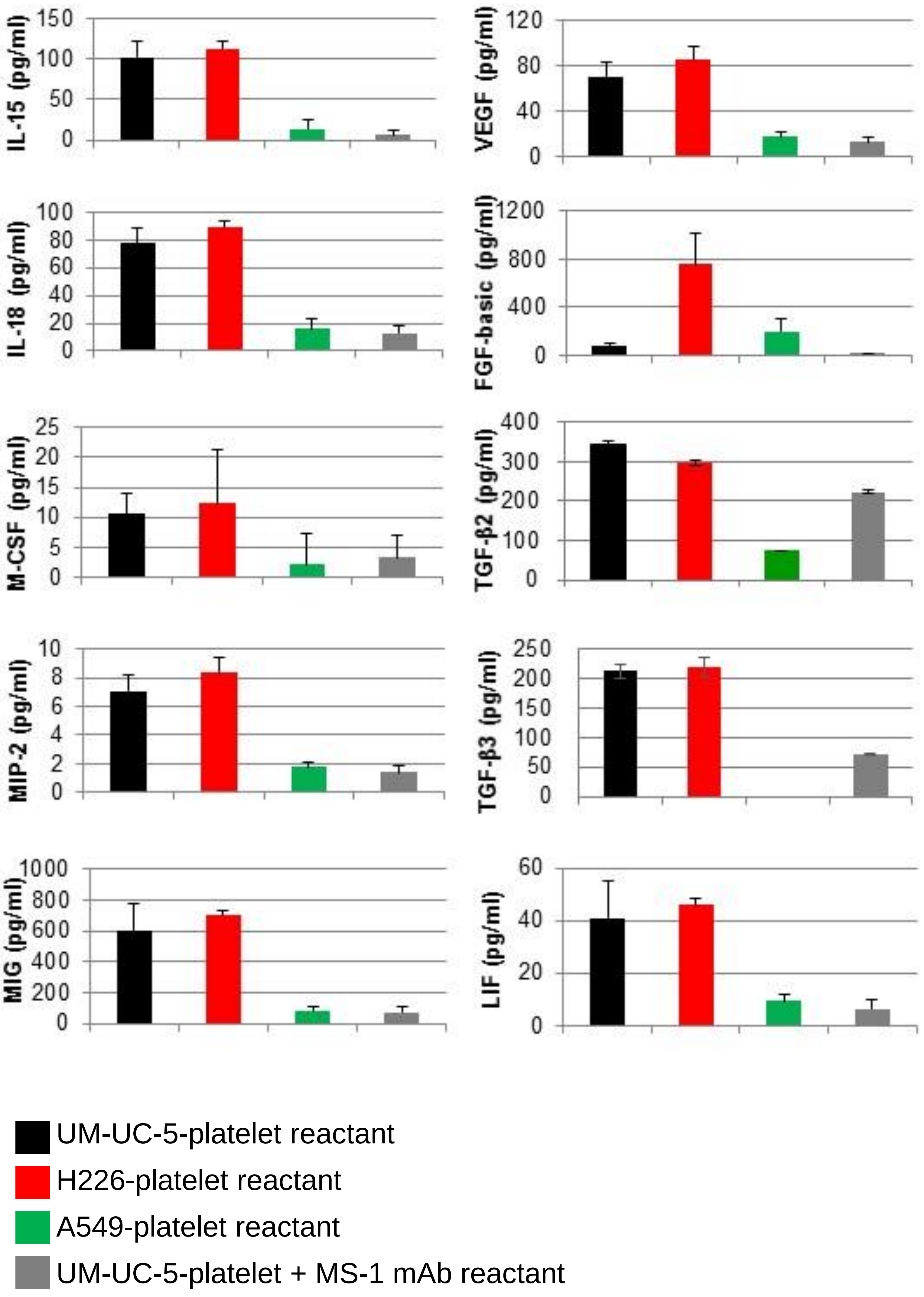

Figure S3. Detection of growth factors and cytokines secreted during podoplanin-induced platelet aggregation by Bio-Plex analyses. UM-UC-5, H226 and A549 cells (5×10^4 cells) were incubated with washed platelets (4×10^7 platelets/200 µl assay scale) suspended in Tyrode's buffer containing 2% platelet-poor plasma and 250 µM $CaCl_2$. In some experiments, UM-UC-5 cells that were pretreated with anti-podoplanin neutralizing mAb MS-1 (10 µg/ml) for 30-40 min on ice were incubated with washed platelets. Concentrations of secreted growth factors and cytokines in the supernatants of tumour-platelet reactants were determined using Bio-Plex multi-cytokine assays (GII 9-Plex and TGF-β 3-Plex). Data (IL-15, IL-18, M-CSF, MIP-2, MIG, VEGF, FGF-basic, LIF, TGF-β2, TGF-β3) except PDGF-BB and TGF-β1 (Fig. 2a) are shown as means ± standard deviation (SD, *n* = 3 or 5).

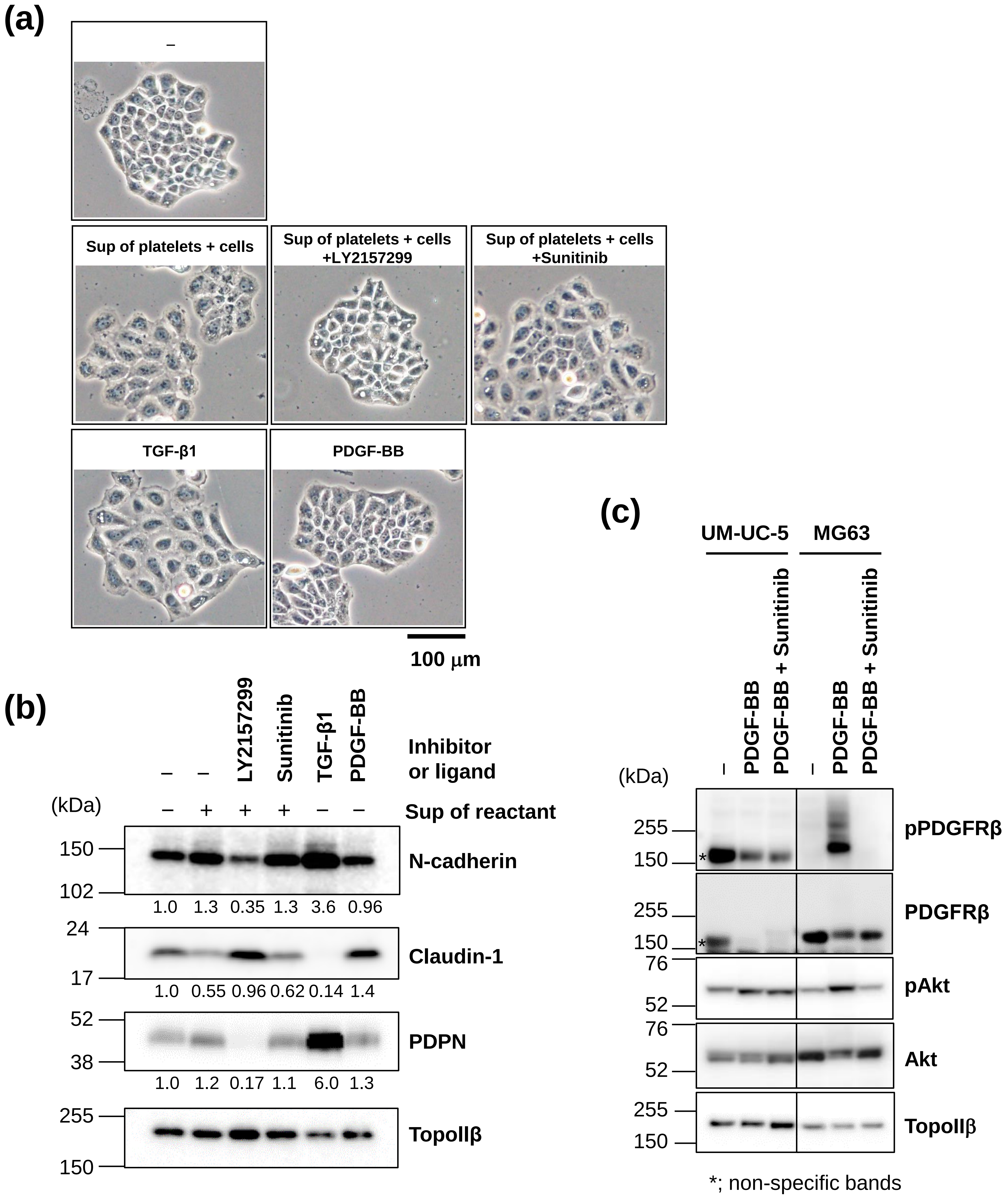

Figure S4. EMT-induction in UM-UC-5 does not depend on PDGFR signalling. (a, b) Supernatant of platelet-UM-UC-5 reactants (2 × 10^8/ml platelets and 2.5 × 10^5/ml cells) was added to UM-UC-5 cells (1 ml of supernatant to 3 ml of medium) treated without or with TGFβR inhibitor (LY2157299, 10 μM) or multi-targeted receptor tyrosine kinase inhibitor (sunitinib, 1 μM, Selleck Chemicals) for 1 h in advance. UM-UC-5 was also treated with TGF-β1 (5 ng/ml) or PDGF-BB (10 ng/ml, R&D Systems). After 48 h of co-culture, cell images were captured by phase-contrast microscopy (a) and immunoblotted with antibodies to N-cadherin, Claudin-1, podoplanin (PDPN), and TopoIIβ (b). The quantified signal of each band in the immunoblots was normalized by TopoIIβ signal, and relative values against the control sample were indicated. (c) UM-UC-5 and MG63 cells (ATCC) treated without or with sunitinib for 1 h in advance were incubated with PDGF-BB (10 ng/ml). After 30 min of incubation, the cells were lysed and immunoblotted with antibodies to phospho-PDGFRβ (Tyr1009) (42F9, Cell Signaling Technology), PDGFRβ (P-20, SantaCruz), phospho-Akt (Ser473) (D9E, Cell Signaling Technology), and Akt (C67E7, Cell Signaling Technology). PDGFR-Akt signal activation was not detected in UM-UC-5, although it was detected and inhibited by sunitinib in MG63.

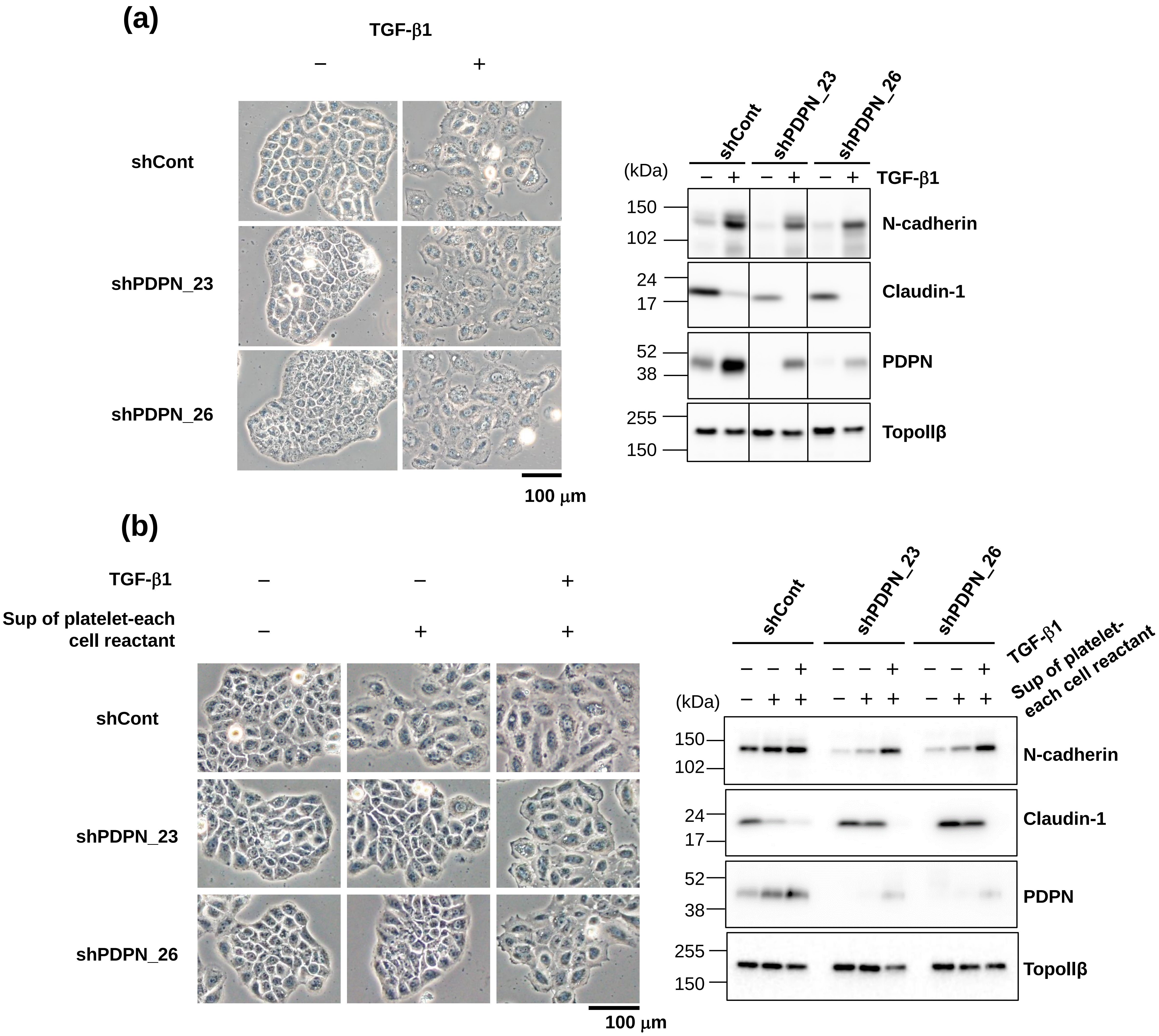

Figure S5. Responsiveness to TGF-β of podoplanin knockdown cells. (a) UM-UC-5 cells transfected control shRNA-expression vector (shControl) or podoplanin-targeting shRNA expression vector (shPDPN_23, 26) were treated without or with TGF-β1 (5 ng/ml) for 48 h. The cell images were captured by phase-contrast microscopy (left) and the cells were lysed and immunoblotted with N-cadherin, Claudin-1, podoplanin, and TopoIIβ antibodies (right). Podoplanin knockdown cells were responsive to TGF-β1 treatment as well as shControl cells. (b) UM-UC-5 shControl and shPDPN cells were co-cultured with the supernatants of platelet-each cell reactants supplied with or without TGF-β1 (3 ng/ml). The morphological and immunoblotted images were shown same as (a). Podoplanin knockdown cells were not induced by treatment with the supernatants of platelet-cell reactants and were rescued by the addition of TGF-β1 to the supernatants.

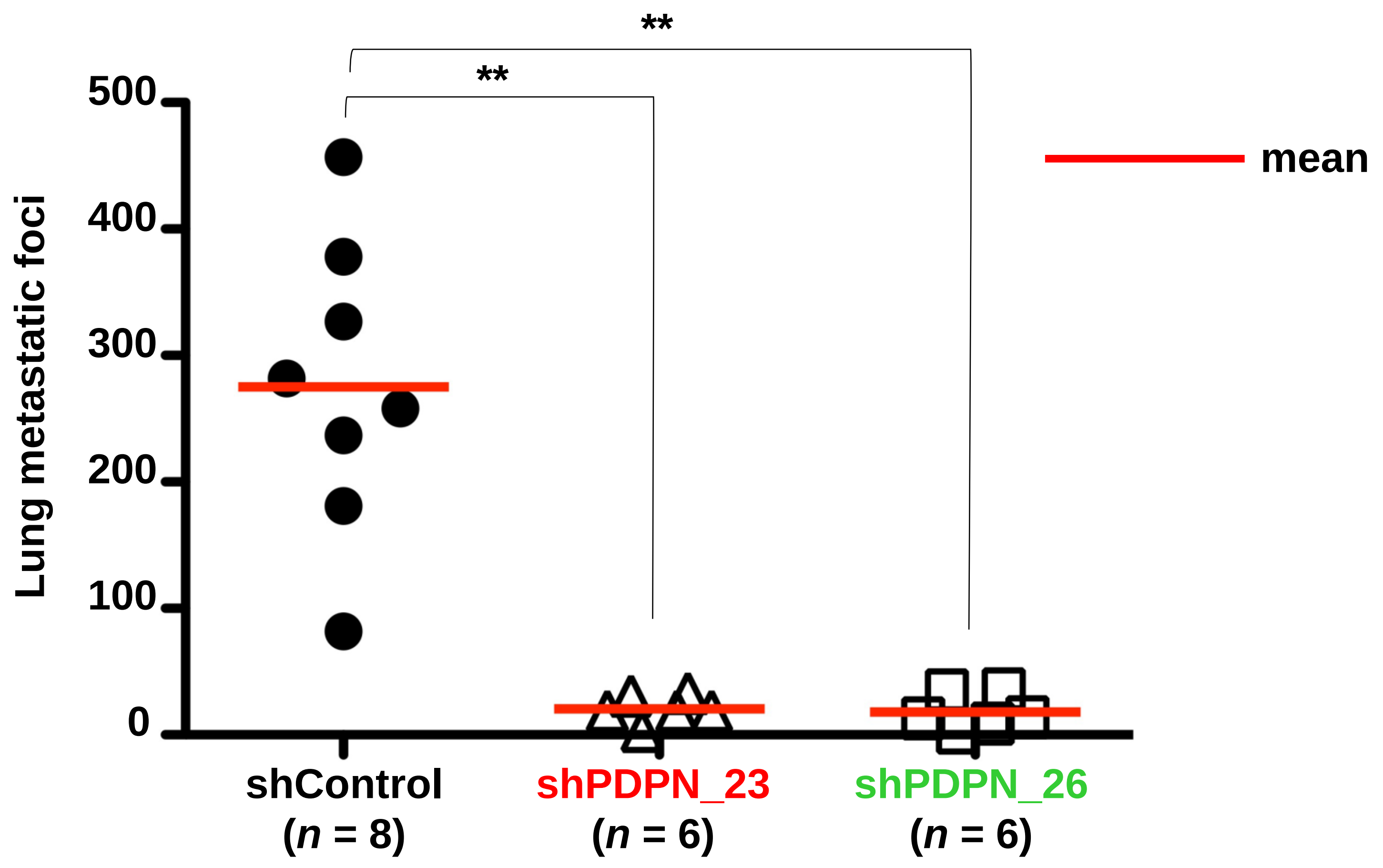

Figure S6. Podoplanin is critical during lung metastasis of UM-UC-5 cells in an *in vivo* model. UM-UC-5 cells were infected with lentivirus containing shRNA targeting human podoplanin (shPDPN_23 and shPDPN_26) or control (shControl). Stable transfectants were then intravenously inoculated into 5-week-old male CB17/Icr-Prkdc^{scid}/CrlCrlj mice. Mice were euthanized 36 days after cell inoculation and metastatic foci on the lung surface were counted. Bars indicate means of the metastatic foci. ***P* <0.01 by the Mann-Whitney *U* test.

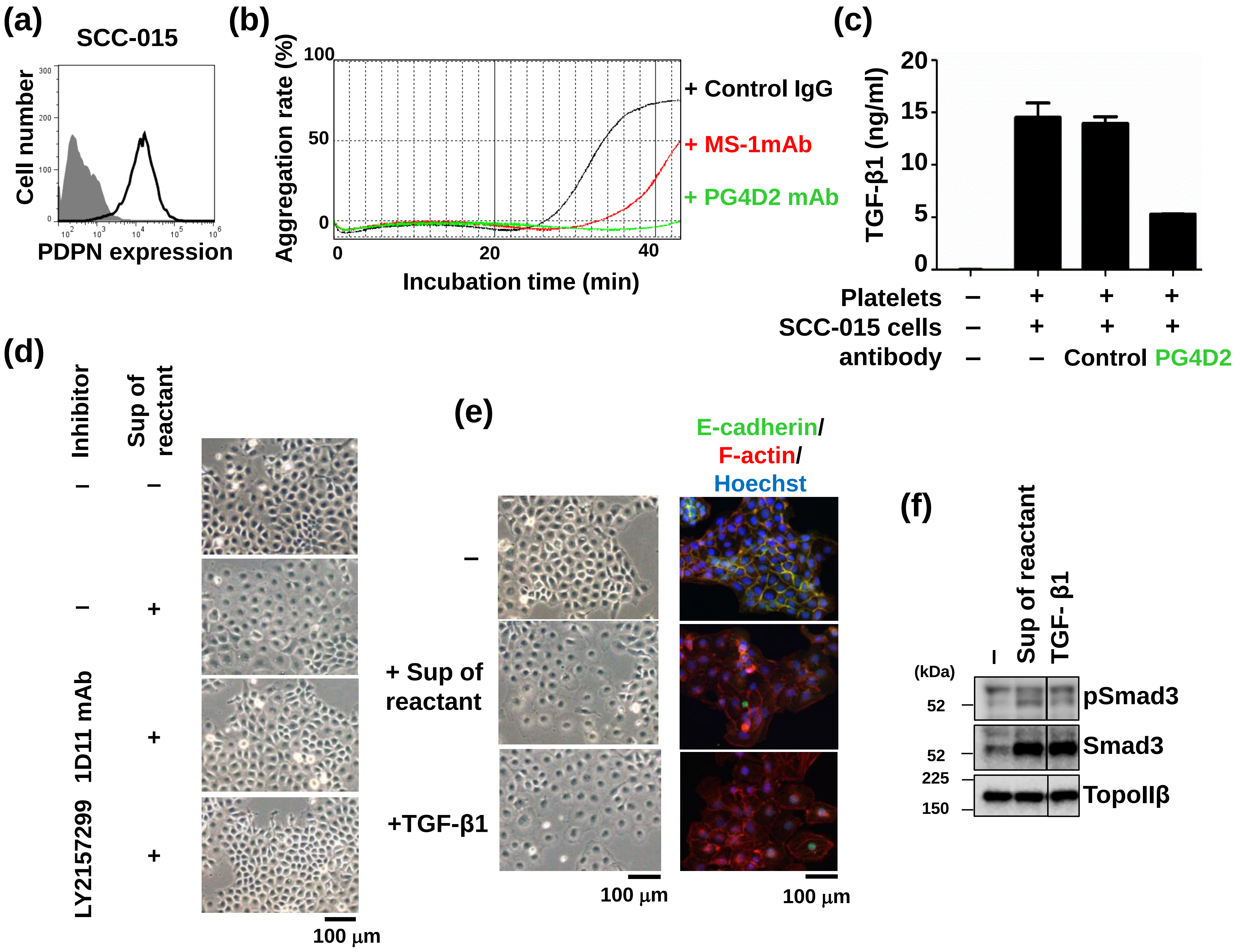

Figure S7. Platelet aggregation, TGF-β release and epithelial-mesenchymal transition in patient-derived podoplanin-positive SCC-015 lung squamous cell carcinoma cells. (a) Flow cytometric analysis of podoplanin expression in established patient-derived SCC-015 lung squamous cell carcinoma cells using anti-podoplanin mAb (D2-40) and Alexa488-conjugated anti-mouse IgG. (b) SCC-015 cells (5×10^4) were treated with mouse control IgG (Control IgG), MS-1 mAb or PG4D2 mAb (100 µg/ml), followed by incubation with platelets (6×10^7/0.2 ml assay) at 37° C. Platelet aggregation was measured using an aggregometer. (c) TGF-β1 concentration in reactants were measured by enzyme-linked immunosorbent assay. All data are shown as means ± standard deviation (SD, n = 3). SCC-015 cells induced TGF-β1 release from platelets, which was suppressed by treatment of the cells with anti-podoplanin neutralizing mAb (clone PG4D2) (100 µg/ml). All data are shown as means ± SD (n = 3). (d) SCC-015 cells were either left untreated (−) or treated with TGF-β neutralizing mAb 1D11 (10 µg/ml) or TGF-βR inhibitor LY2157299 (10 µM) for 2 h. Cells were then incubated with supernatants of PBS (−) or SCC-015-platelet reactants (+) for 48 h. Morphological changes were observed under phase-contrast microscopy. Scale bars indicate 100 µm. (e, f) SCC-015 cells were either left untreated (−) or treated with supernatants of SCC-15-platelet reactants or TGF-β1 (3 ng/ml) for 48 h. (e) Morphological changes were observed under phase-contrast microscopy (left panels). Cells were stained for E-cadherin (green), F-actin (red; phalloidin) and nuclear DNA (blue; Hoechst 33342). Physiological changes of cells were observed under immunofluorescence microscopy (right panels). Scale bars indicate 100 µm. (f) Cell lysates were immunoblotted with antibodies against phospho-Smad3 (pSmad3), Smad3 and TopoIIβ.

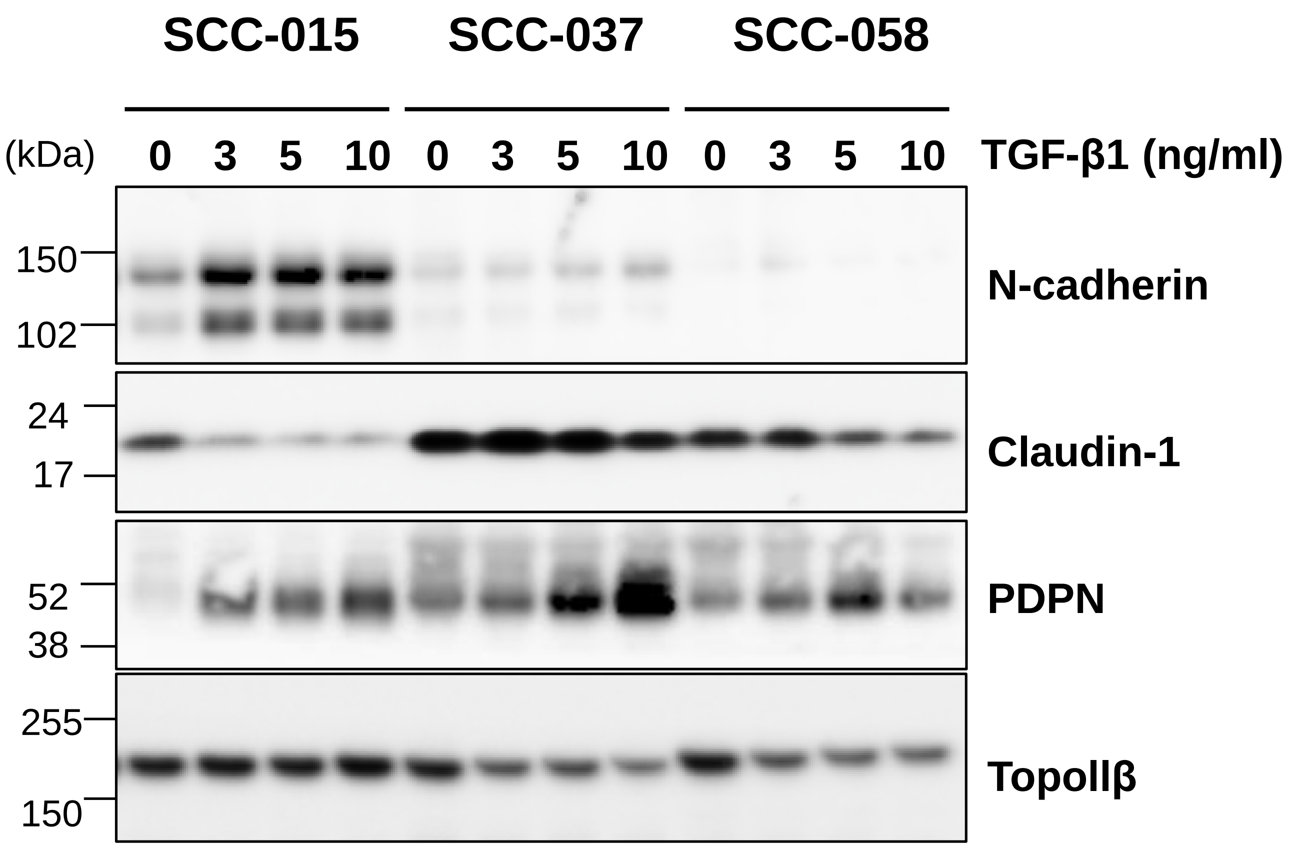

Figure S8. Responsiveness to TGF-β treatment of established lung squamous cell carcinoma cells from clinical samples. Podoplanin-positive lung squamous cell carcinoma cells, SCC-015, -037, and -058, were cultured without or with TGF-β1 (3, 5, and 10 ng/ml) for 48 h. The cells were lysed and immunoblotted with antibodies to N-cadherin, Claudin-1, podoplanin (PDPN), and TopoIIβ. SCC-015, which has the potential to metastasize in the hematogenous metastasis mouse model, were EMT-induced by TGF-β1, but SCC-037 and SCC-058, which are hard to metastasize, showed weak responsiveness to TGF-β1.

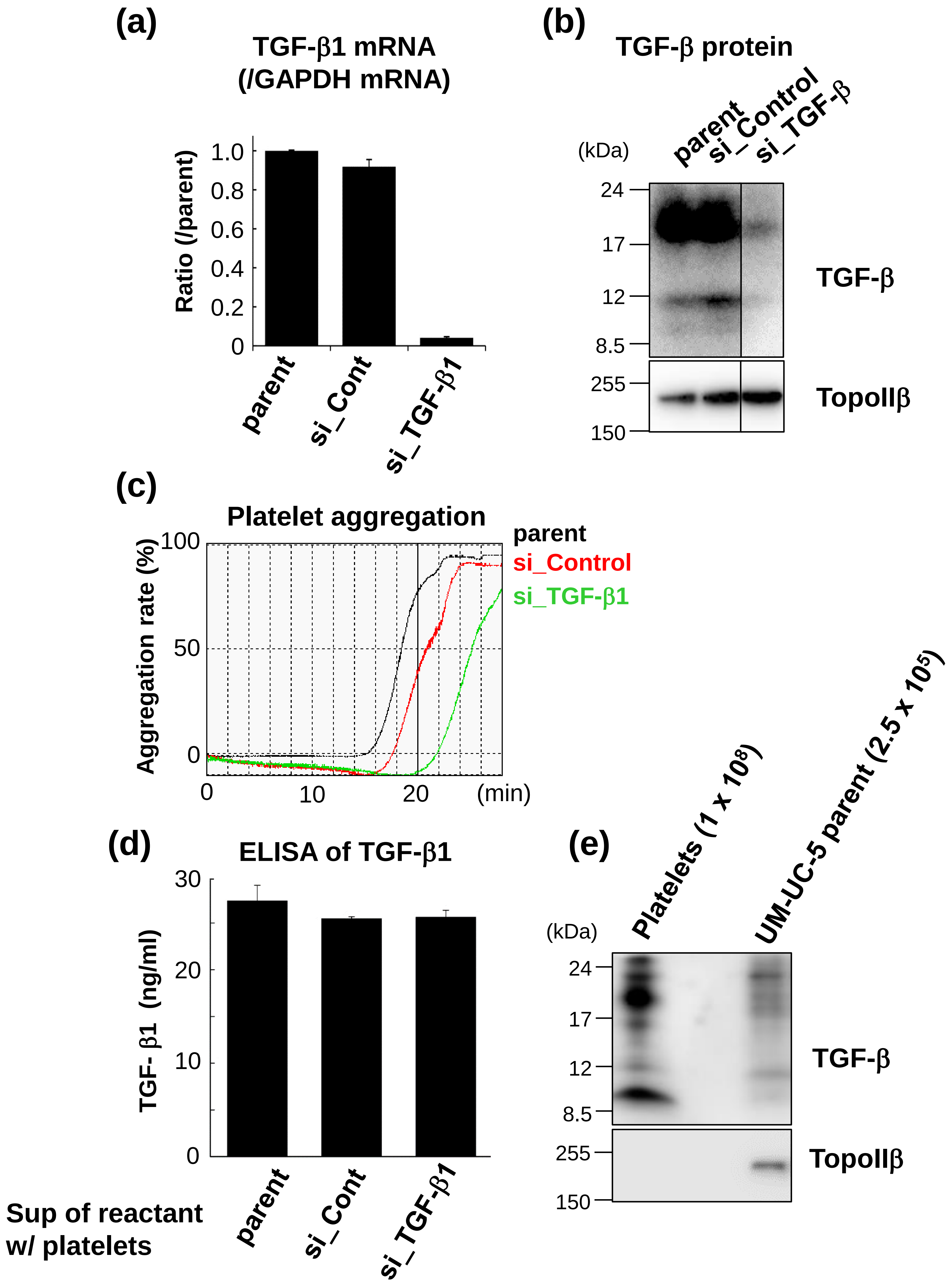

Figure S9. Released TGF-β in supernatant of UM-UC-5 cell-induced platelet aggregation is derived from platelets. (a-d) TGF-β1 in UM-UC-5 was knocked down using siRNA (ON-TARGETplus Human TGFB1 (7040) siRNA SMARTpool; si_TGF-β1, and Non-targeting Pool; si_Control, Dharmacon). At 48 h after transfection of 20 nM siRNA, UM-UC-5 cells were collected for analysis. (a) Knockdown of TGF-β1 mRNA was confirmed by RT-qPCR. Means ± SD (*n* = 3) are indicated. The primer sequences used were 5′-TTGATGTCACCGGAGTTGTG-3′ and 5′-TGATGTCCACTTGCAGTGTG-3′ for tgfb1, and TCAGCCGCATCTTCTTTTGC and 5′-TTAAAGCAGCCCTGGTGAC-3′ for gapdh. (b) TGF-β1 knockdown was confirmed by immunoblotting with anti-TGF- β mAb (1D11). TopoII β was detected as a loading control. (c) Parent and siRNA-transfected UM-UC-5 cells (1 × 10^5) were incubated with platelets (4 × 10^7/0.2 ml assay), and the aggregation rate was monitored by an aggregometer. (d) The reactants of (c) were centrifuged and the TGF-β1 concentration was examined by ELISA. Means ± SD (*n* = 3) are indicated. (e) TGF-β1 contained in platelets and UM-UC-5 cells was compared by immunoblotting. Samples were loaded at the same ratio in platelet aggregation analysis (c); platelet : UM-UC-5 cell ≈ 400 : 1.

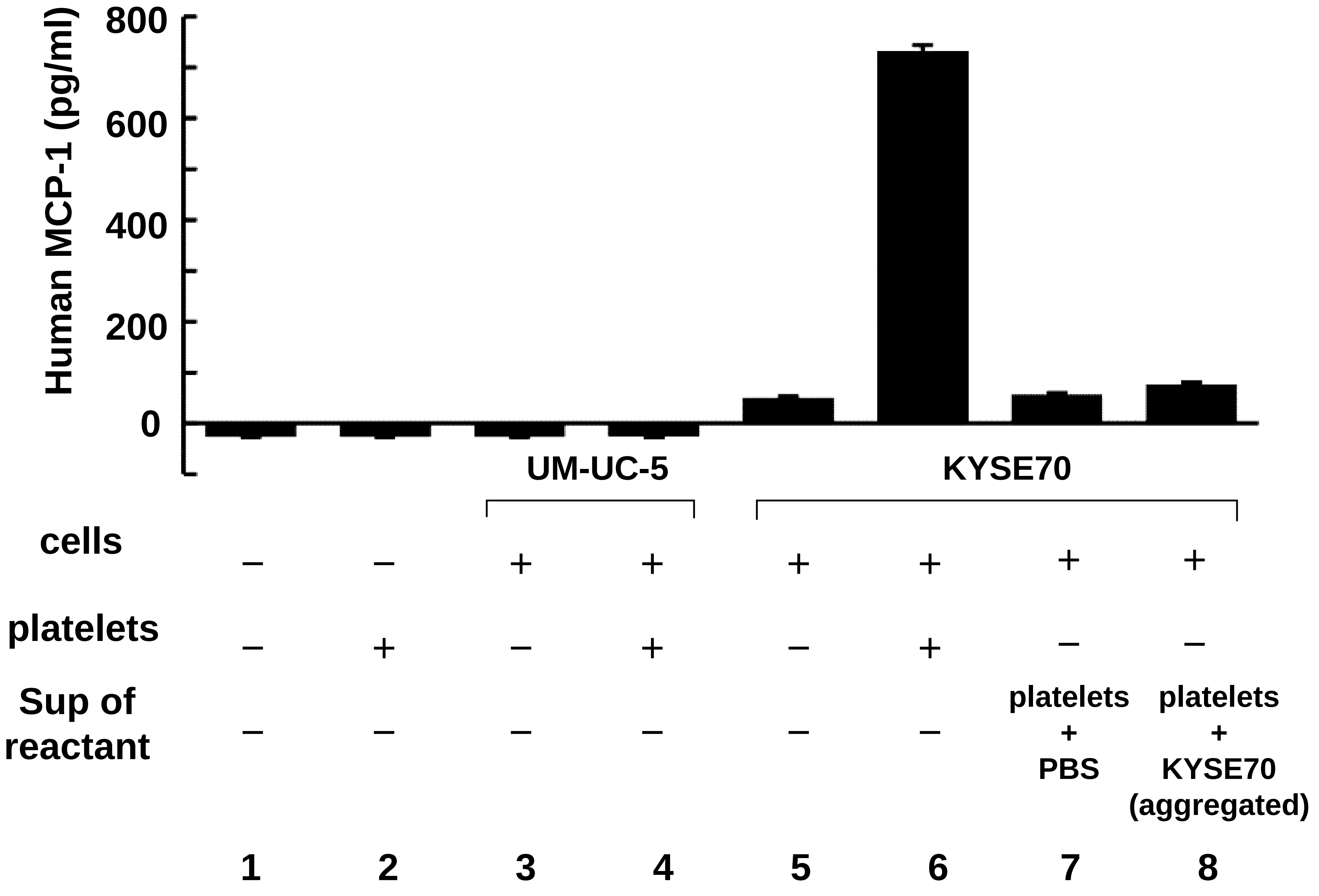

Figure S10. NF-κB signalling in UM-UC-5 is not activated through direct contact with platelets. MCP-1 released from tumor cells was detected by ELISA as an indication of NF-κB signal activation. UM-UC-5 (3, 4) or esophageal SCC cells, KYSE70 (JCRB) (5–8) cultured in 12-well plates with 1 ml of medium per well were added with 250 μl of control Tyrode's buffer (1, 3, 5), platelets (10^9/ml) (2, 4, 6), or supernatant of platelet reactant with control PBS (7) or KYSE70 cells (8). After 20 h co-culture, each medium was collected and centrifuged. The supernatants were analyzed using Human CCL2/MCP-1 Quantikine ELISA Kit (R&D SYSTEMS). Means ± SD (n = 3) are indicated.

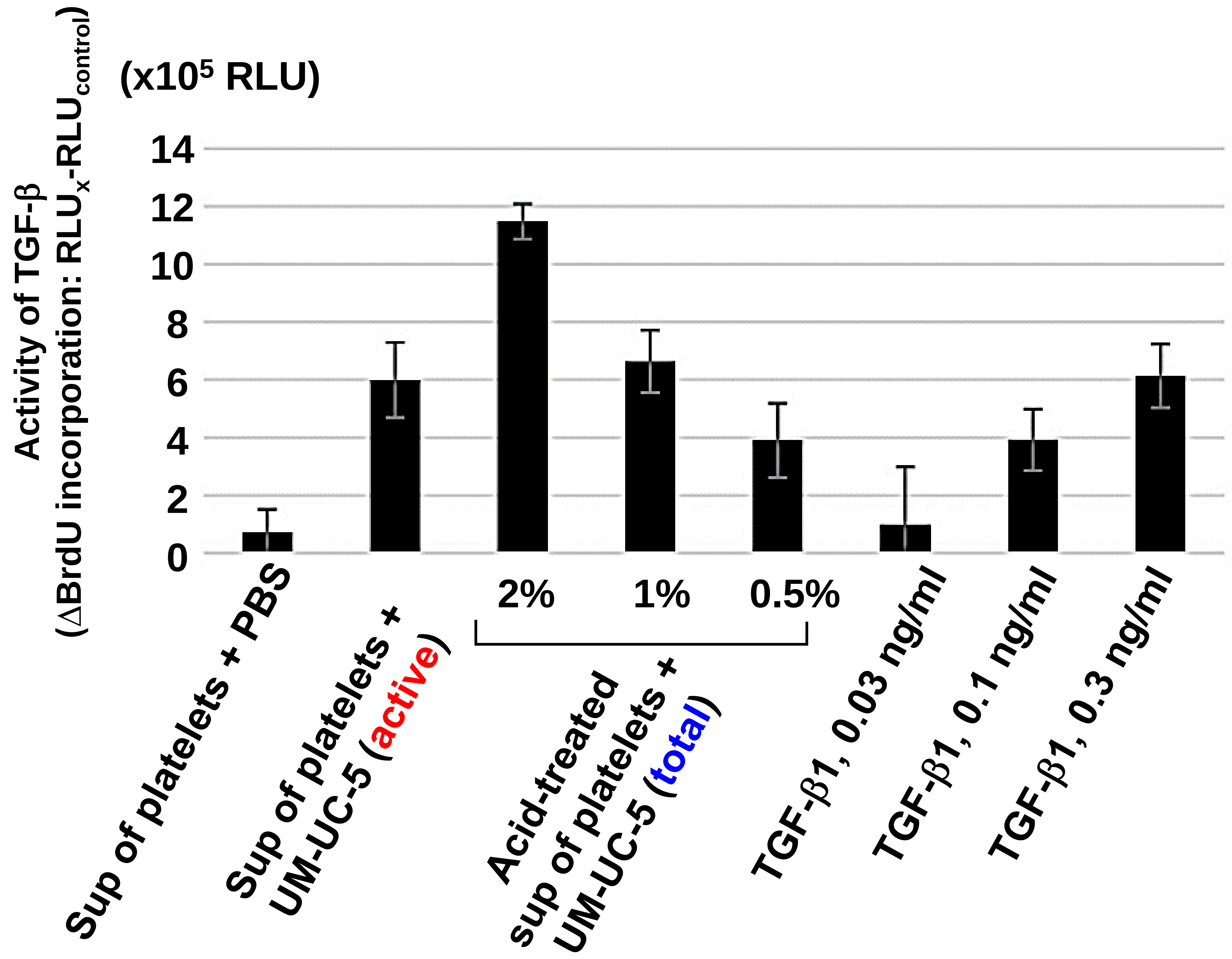

Figure S11. Detection of active TGF-β in supernatant of platelet reactant by bioassay. Active TGF-β was detected as growth suppression activity of Mink lung cell line (Mv1Lu, ATCC). Growth was analyzed by detection of BrdU incorporation with ELISA system (Cell Proliferation ELISA, BrdU, chemiluminescent, Roche). Mv1Lu was seeded (3×10^3/100 μl/well of 96-well plate) and added 50 μl of the supernatant of platelet-cell reactant or TGF-β1 to the culture on the next day. After 22 h of incubation, BrdU was added and incorporated for 2 h and analyzed by ElISA. Growth suppression was indicated as the growth distance compared with that of control cells which was added only control Tyrode's buffer to the culture. Means ± SD (n = 3) are indicated. Supernatant of the reactant was prepared using 8×10^8/ml platelets and 4×10^6/ml UM-UC-5 or PBS. For detection of total TGF-β, the supernatant was acid-treated; 20 μl of 1 N HCl was added to 100 μl of supernatant, incubated for 10 min at room temperature, and neutralized by adding 20 μl of 1.2 N NaOH /0.5 M HEPES.

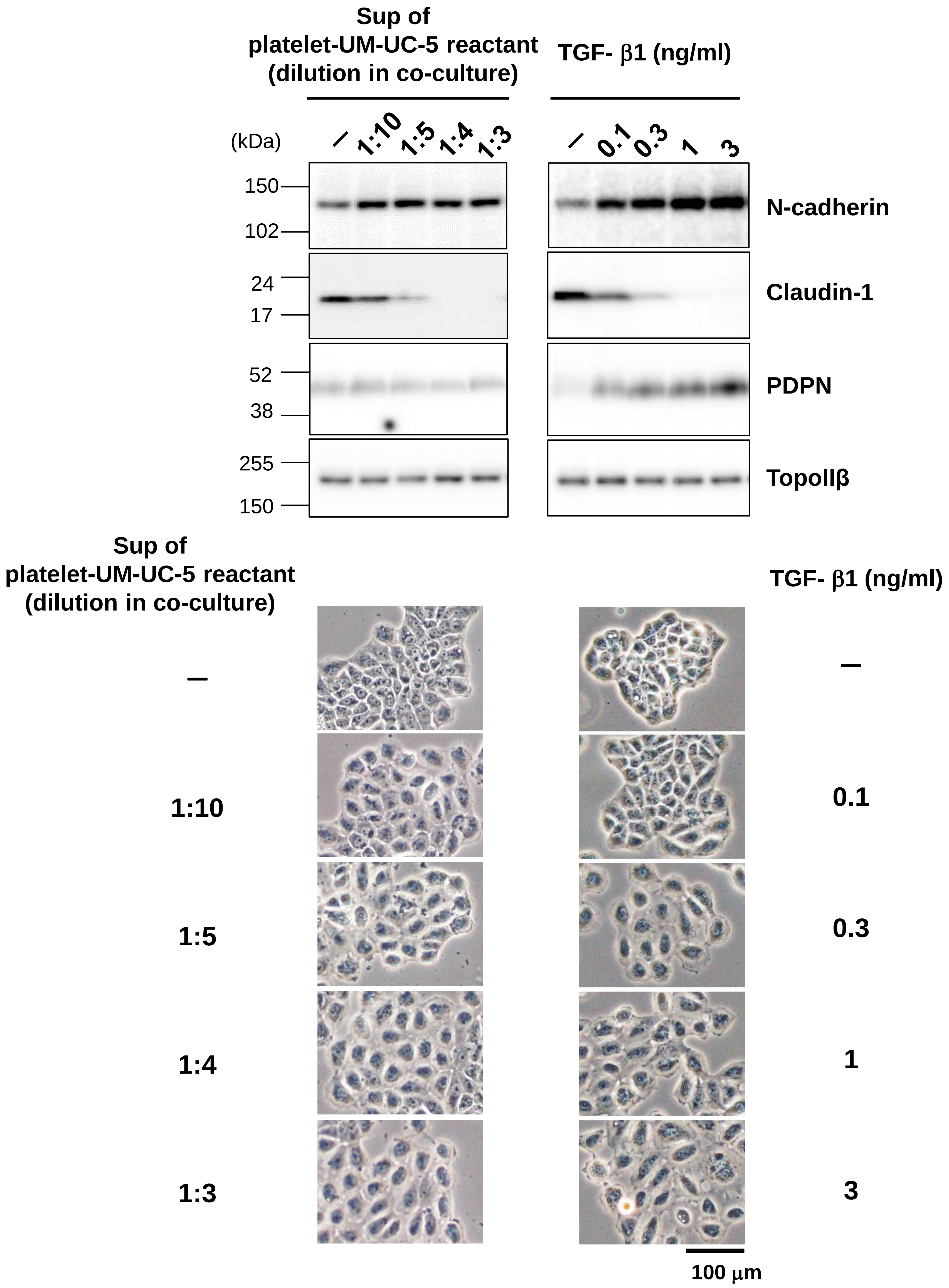

Figure S12. Required concentration of TGF-β or supernatant for EMT induction in UM-UC-5. UM-UC-5 cells were incubated with dilution series (1:10, 1:5, 1:4, and 1:3) of supernatant of the platelet-cell reactant (2.5 × 10^8 /ml platelets) or TGF-β1 (0.1, 0.3, 1, and 3 ng/ml). After 48 h of incubation, the cell lysates were immunoblotted with antibodies to N-Cadherin, Claudin-1, podoplanin (PDPN), and TopoIIβ (upper). Morphological images of the cells were captured by phase-contrast microscopy (lower).

(a)

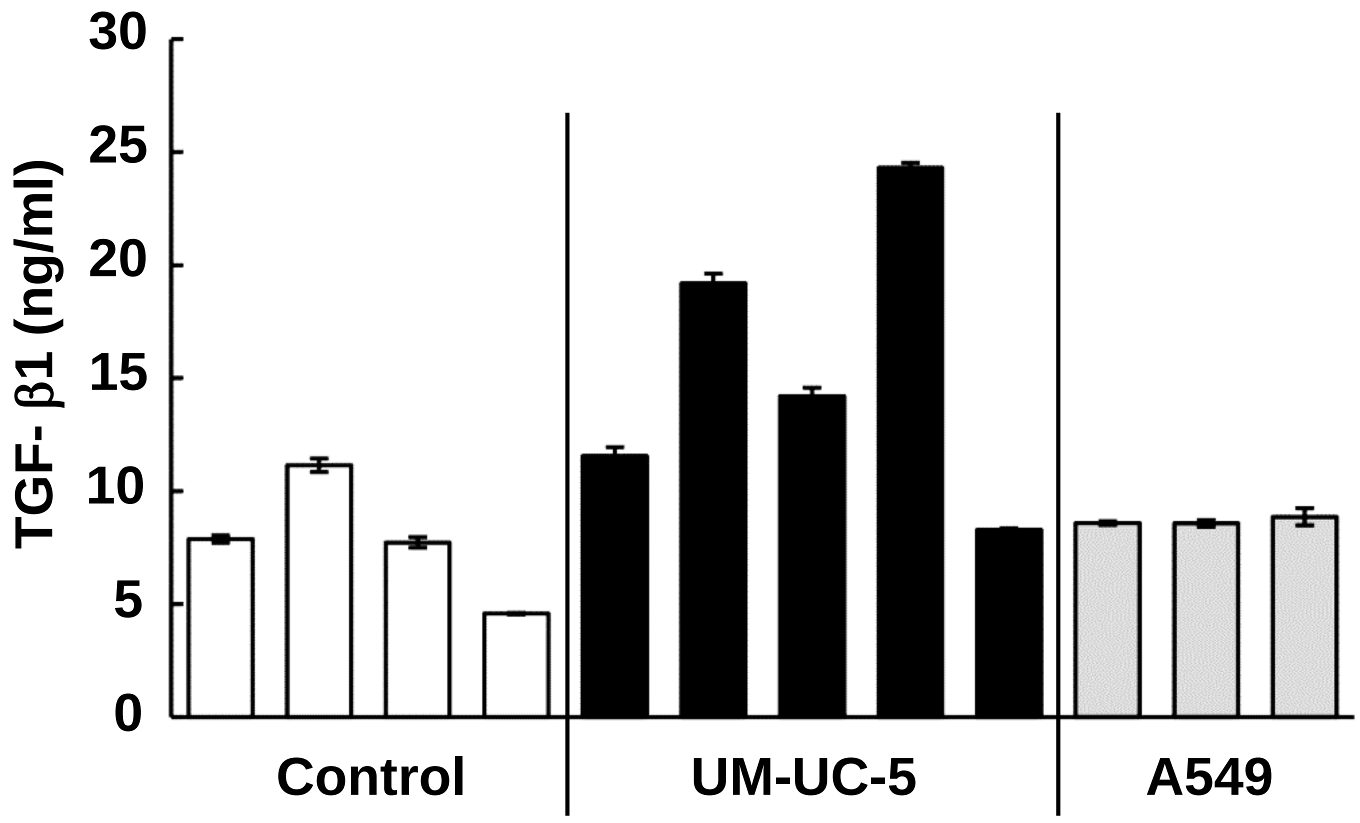

(b)

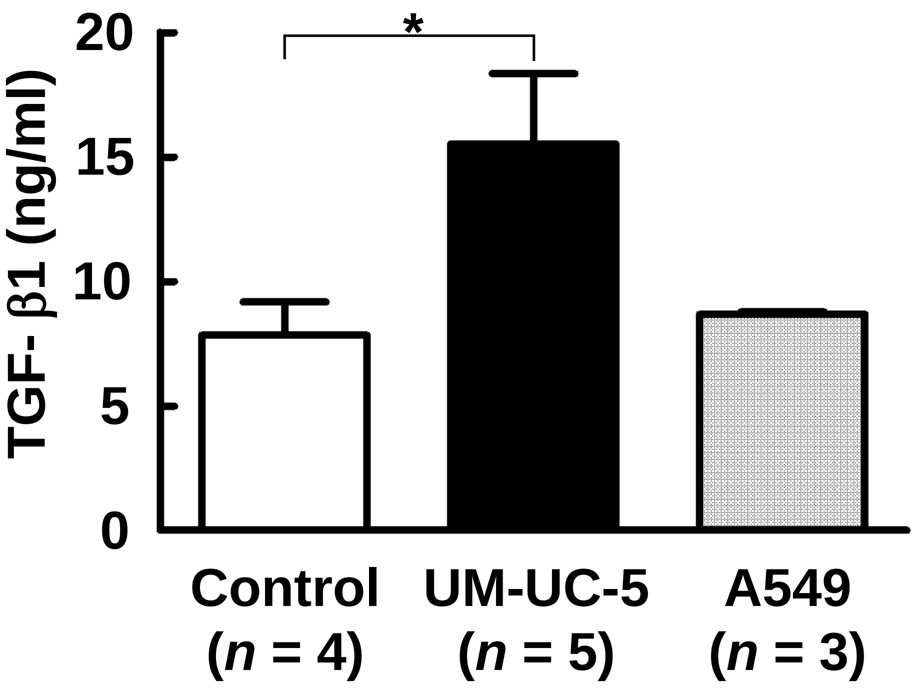

Figure S13. Concentration of TGF-β1 in blood is increased after intravenous injection of UM-UC-5 cells. After intravenous injection of control PBS, podoplanin-positive UM-UC-5, or podoplanin-negative A549 cells (1×10^6/mouse) into the tail vein of ICR mice, blood plasma was prepared from whole blood drawn with heparin within 1 h. (a) The concentration of TGF-β1 in each plasma sample was analyzed by ELISA. Means ± SD ($n = 3$) are indicated. (b) From data in (a), the calculated means ± SEM are shown. *$P < 0.05$ by the Mann–Whitney U test. $P = 0.0275$.
